# Protecting quantum Fisher information of *N*-qubit GHZ state by weak measurement with flips against dissipation

**DOI:** 10.1038/s41598-017-04726-1

**Published:** 2017-07-21

**Authors:** Yu Chen, Jian Zou, Zheng-wen Long, Bin Shao

**Affiliations:** 1 School of Physics, Beijing Institute of Technology, Beijing, 100081 China; 2 School of Physics and Electronic science, Guizhou Normal College, Guiyang, 550018 China; 3 College of Physics, Guizhou University, Guiyang, 550025 China

## Abstract

In this paper we propose a scheme by using weak-measurement-based pre- and post-flips (WMPPF) to protect the average quantum Fisher information (QFI) in the independent amplitude-damping channel (ADC) for *N*-qubit GHZ state and generalized *N*-qubit GHZ states. We also discuss the weak measurement and quantum measurement reversal (WMQMR) with the same ADC. Based on the analytical and numerical results we obtain the main result: the WMPPF can reduce the effect of dissipation on the average QFI of the phase or the frequency for GHZ state and some generalized GHZ states, and the WMQMR can reduce the effect of dissipation on the average fidelity for GHZ state and generalized GHZ states in ADC. Comparing QFI with fidelity for WMPPF or for WMQMR, a scheme protecting the average fidelity does not necessarily protect the average QFI, even with the same parameters, and vice versa. We also focus on the average QFI versus *N* in the phase estimation and the frequency estimation of WMPPF, both of which show the advantages over the do-nothing (DN) case. From the investigation of the QFI of weight factor, we find that increasing qubit number can protect it both for WMPPF and for DN.

## Introduction

Quantum metrology offers a significant advantage over classical approaches, where the usage of quantum entanglement leads to an improved scaling in the achievable precision in parameter estimation^1, 2^. The attainable precision *δϕ* is lower-bounded by the quantum Cramér-Rao bound^3^ $\delta \varphi \ge 1/\sqrt{\nu F}$ with *ϕ* the parameter to be estimated, where *F* is the QFI and *ν* is the measurement times. Therefore the theoretical acquirable estimation precision is determined by quantum Fisher information (QFI)^4–6^. So how to improve the QFI is an important task in quantum metrology. However, for any practical application, i.e., in open quantum system, the inevitable impact of decoherence needs to be taken into account in order to correctly quantify the ultimate attainable gain in precision. There are several ways to overcome the obstacle of decay caused by the noise channel in quantum metrology such as: using dynamical decoupling to improve the scaling in noisy quantum metrology^7, 8^, using spin squeezing which can lead to a significant reduction of spin noise, and hence an increase in magnetometric sensitivity^9^, using quantum error correction in both phase and frequency estimation^10, 11^, using external ancillae and adapting the classical simulation and finite-N channel extension methods to optimize the duration of evolve-and-measure rounds^12–14^, using decoherence free subspaces with Ramsey interferometry in the presence of collective dephasing which can significantly enhances the precision^2^, using weak value amplification and postselection^15, 16^, and using weak measurement (WM)^17–21^ in feedback control^22^.

The important consequence of the physical nature of measurement is the so-called quantum back-action which extract the information by WM from a system can give rise to a feedback^19, 23–25^ effect in which the system configuration after the measurement is determined by the measurement outcome. The WM with an optimum measurement strength which achieves the best trade-off between gaining information about the system and disturbing it through measurement back-action. It is found that the optimal recovery from noise for the system can be realized^18^, and the quantum control schemes based on WM with appropriate measurement strengths can realize the optimal protection from the noise^17, 26, 27^. We are interested in how to use WM to protect the average QFI of *N*-qubit GHZ state^28–30^ against dissipation^1, 31, 32^ in this paper. And we propose a new scheme which may protect the QFI and average fidelity of different multipartite entanglement systems from the noises of amplitude-damping channel (ADC)^33, 34^. And this scheme uses WM with pre- and post-flips (WMPPF). We focus on the protecting precision of phase estimation^35–37^ against dissipation^2, 38^ for *N*-qubit GHZ state where the phase sensitivity can achieve the Heisenberg limit^39–43^ at the beginning. For comparison, we discuss another scheme^17^ that uses weak measurement and quantum measurement reversal (WMQMR).

We will display the evolved average QFI and the average fidelity of WMPPF, WMQMR and do-nothing (DN) case (i.e., do nothing with the ADC), which shows that the WMPPF scheme has the advantage in the average QFI and sometimes has advantage in the average fidelity for *N*-qubit GHZ state. The WMQMR has advantage in average fidelity to GHZ state, but not uselful in average QFI to GHZ state. We also focus on the average QFI versus *N* in the phase estimation and the frequency estimation, and both the phase and the frequency estimations of WMPPF show the superiority. Our scheme has advantages not only in GHZ state but also in a lot of generalized GHZ states, i.e., our scheme is independent of the concrete coefficients (or weight parameter) of some generalized GHZ states in the QFI protecting. By comparing QFI and fidelity for any of the two schemes with the same *N* and the magnitude of the decoherence, we can get a conclusion that when QFI is high the fidelity is not always high and vice versa. And at last, we investigate the QFI of the weight factor for WMPPF, WMQMR and DN. The calculations infer that WMPPF can only protect the average QFI to some generalized GHZ state when time is not small, and the average QFI of WMQMR case is always below the QFI of DN case, and DN can get better QFI as the number of the qubits increases for GHZ state or generalized GHZ state. This character of DN to us means that an feasibility of resisting the decay of ADC.

## Results

### The schemes

**Figure 1:**
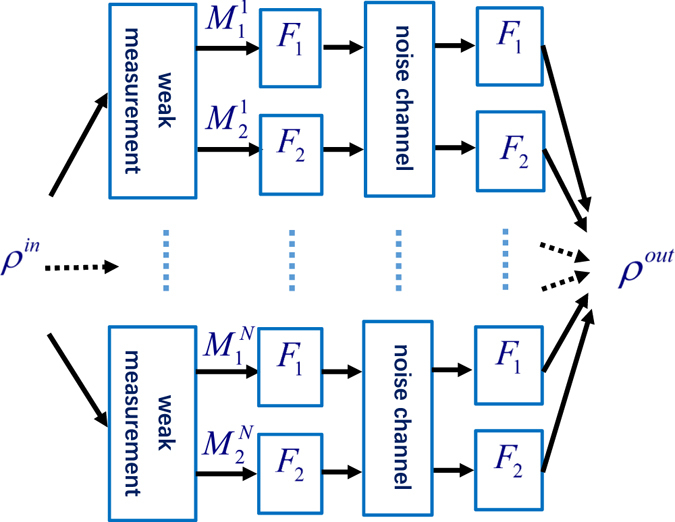
WMPPF scheme for *N*-qubit state.

In what follows, we will mainly discuss our scheme. The WMPPF is shown in Fig. 1. This process is like this: Before the noise channel the WM is made and according to different measurement results the operations of pre-flips are applied in order to transform the protected state to some state, and after the individual noise channel ADC one can use post-flips with the state. At last we can get the evolved state. The initial state is chosen to be a *N*-qubit GHZ state of which has past the phase gates and the phase sensitivity can achieve the Heisenberg limit at the beginning. Our calculations of the average QFI and average fidelity are based on the evolved state after our processing. Such a procedure in this case means protecting the average QFI while not direct protecting the state. The estimation precision of phase is protected and will be higher than the DN case. Details of calculations can be found in Additional information.

In this paper, we only investigate the dissipation process: ADC. For each qubit, the Kraus operators of the ADC are refs 13, 17 and 33 $${E}_{1}=(\begin{array}{cc}1 & 0\\ 0 & \sqrt{s}\end{array})$$, $${E}_{2}=(\begin{array}{cc}0 & \sqrt{r}\\ 0 & 0\end{array})$$, where the magnitude of the decoherence $r\equiv 1-s$ represents the probability of decay from the upper level |1〉 to the lower level |0〉, with *s* = *e* ^−Γ*t*^, and Γ is the energy relaxation rate and *t* is the evolving time.

In this paper, we assume that the *N* qubits go respectively through *N* independent quantum channels with different parameters. However, we also want to discuss a simplified version where the *N* qubits have the same parameters: the same WM strength the same reversing measurement strength and the same the magnitude of the decoherence, etc. And in the following discussion, we use $\mathop{=}\limits^{S}$ to indicate this simplified version from the general version.

#### Weak-measurement-based pre- and post-flips

Now we proceed to analyze the scheme WMPPF in detail. We consider a *N*-qubit state (labeled by $1,\ldots ,N$) quantum system whose initial state is generalized GHZ state $|{\rm{\Phi }}\rangle =\,\cos \,(\theta \mathrm{/2)}{|0\rangle }^{\otimes N}$ + ${e}^{i{\varphi }_{0}}\,\sin \,(\theta /2){|1\rangle }^{\otimes N}$, 0 < *θ* < *π*, and *ϕ*_0_ is the initial phase. And *θ* = *π*/2, *ϕ*_0_ = 0 means GHZ state. Without loss of generality, for *N* independent ADCs we suppose that the phase is encoded onto its basis by each of these *N* unitary phase gates: ${U}_{\varphi }=|0\rangle \langle 0|+{e}^{i\varphi }|1\rangle \langle 1|={e}^{-i{\sigma }_{z}\varphi \mathrm{/2}}$, i.e., we choose each of these *N* phase gates acting on each of the qubits^13, 41, 44–48^. Then we can get the input state as refs 2, 36 and 49 $|{{\rm{\Phi }}}^{in}\rangle =\alpha {|0\rangle }^{\otimes N}+\beta {|1\rangle }^{\otimes N}$ which we depict in Fig. 1 as *ρ* ^*in*^, here *α* = cos(*θ*/2), $\beta ={e}^{i(N\varphi +{\varphi }_{0})}\,\sin \,(\theta \mathrm{/2)}$ and it can get the Heisenberg limit if *θ* = *π*/2 at the beginning, where *ϕ* is the phase to be measured^50^. In following we will see that all the derived equations for use are dependent on |*β*|, while they do not rely on *β*, so that we need not to consider the concrete values of *ϕ* and *ϕ*_0_ in the discussing of this paper (i.e., any values for them have the same result for the average QFI, fidelity and probability).

As Fig. 1 depicts the beginning of the processing, We first use two WM operators to measure each qubit of the *N*-qubit generalized GHZ state:

1 $${M}_{1}(p)=(\begin{array}{cc}\sqrt{p} & 0\\ 0 & \sqrt{1-p}\end{array}),\,{M}_{2}(p)=(\begin{array}{cc}\sqrt{1-p} & 0\\ 0 & \sqrt{p}\end{array}).$$

Then we use ${F}_{1}^{i}$ and ${F}_{2}^{j}$ acting on the qubits according to the two different measurement results as shown in Fig. 1. ${F}_{1}^{i}={I}^{i}$ and ${F}_{2}^{j}={\sigma }_{x}^{j}$ represent identity unitary operator and flipping operator according to the *i* and *j* of the solution of ${M}_{1}^{i}$ and ${M}_{2}^{j}$ of the WM, respectively. Here we have $i\in {\mathbb{A}}$, $j\in {\mathbb{B}}$, ${\mathbb{A}}\cap {\mathbb{B}}=\varnothing$ and ${\mathbb{A}}\cup {\mathbb{B}}=\{1,\ldots ,N\}$, where ${\mathbb{A}}$ (or ${\mathbb{B}}$) indicate a concrete combination according to the solution of the WM (details are in the Additional information). Then, the *N* qubits pass through the ADC. After the ADC, *F*_1_ or *F*_2_ are used again on each qubit, the same as those used before they pass into the ADC. That is to say, if at first the measurement is *M*_1_, then before the ADC we use *F*_1_, and after ADC we still use it. Or if at first the measurement is *M*_2_, then before and after the ADC we will use *F*_2_.

From processing of the scheme WMPPF in Fig. 1, at last, we can get the evolved normalized matrix *ρ* ^*out*^. The processing has 2^*N*^ kind results of *ρ* ^*out*^ which come from the 2^*N*^ kind measurement results of the WM. Although the results of *ρ* ^*out*^ is different, they have similar structure which can give us the chance to calculate their average QFI and average fidelity analytically. Because the WM with the pre- and post-flips are complete, this scheme always has a probability of 1, which also can be testified from Eq. (12) of Additional information. The average QFI of WMPPF then is (see Additional information for detailed calculations)

2 $$\begin{array}{lll}{F}_{WMPPF} & \mathop{=}\limits^{S} & \frac{4{|\alpha \beta |}^{2}{N}^{2}{p}^{N}{\mathrm{(1}-p)}^{N}{s}^{N}}{{|\alpha |}^{2}{\mathrm{((1}-p)s)}^{N}+[{|\alpha |}^{2}{\mathrm{((1}-p)r)}^{N}+{|\beta |}^{2}{p}^{N}]}\\  &  & +\frac{4{|\alpha \beta |}^{2}{N}^{2}{p}^{N}{\mathrm{(1}-p)}^{N}{s}^{N}}{[{|\alpha |}^{2}{p}^{N}+{|\beta |}^{2}{\mathrm{((1}-p)r)}^{N}]+{|\beta |}^{2}{\mathrm{((1}-p)s)}^{N}}\\  &  & +{\sum }_{k=1}^{N-1}\,{{\mathbb{C}}}_{N}^{k}\frac{4{|\alpha \beta |}^{2}{N}^{2}{p}^{N}{\mathrm{(1}-p)}^{N}{s}^{N}}{{|\alpha |}^{2}[{p}^{k}{\mathrm{((1}-p)s)}^{N-k}]+{|\beta |}^{2}[{\mathrm{((1}-p)s)}^{k}{p}^{N-k}]}.\end{array}$$

Above if we suppose *θ* = *π*/2, including GHZ state, and *ϕ, ϕ*_*0*_ can be any value. However, from this expression, we can see that the average QFI of WMPPF is independent with *ϕ*. And from this expression, if we divide the numerator and the denominator with *s* ^*N*^ on each fraction of the right side of the equal sign, we can find that average QFI get the maximum for any *N* when *r* = 0 (i.e., *s* = 1).

We can also get the average fidelity of WMPPF (see the Additional information for detailed calculations):

3 $$\begin{array}{ccc}Fi{d}_{WMPPF} & \mathop{=}\limits^{S} & \frac{1}{{P}_{WMPPF}}\{{|\alpha |}^{4}(p+(1-p)s{)}^{N}+2|\alpha \beta {|}^{2}{(1-p)}^{N}{r}^{N}\\  &  & +{|\beta |}^{4}{(p+(1-p)s)}^{N}\\  &  & +{2}^{N+1}{|\alpha \beta |}^{2}{(sp(1-p))}^{N/2}\}.\end{array}$$

It can be easily found from Eqs (2) and (3) that for *r* = 0 and *p* = 1 − *p* the average QFI and the average fidelity can get the maximums *N* ^2^ and 1 for *GHZ* state, respectively. And from this we can conclude that when *r* = 0, *p* = 1/2 is the optimal *p* value both for average QFI and average fidelity. And this condition can help us to choose *p* to draw the start point (i.e., at *r* = 0) of the curve evolving with *r* where the average QFI and the average fidelity begin to evolve from the maximal values. However, this condition can not help to get the maximal average QFI and the maximal average fidelity while *r* > 0, and for *r* > 0 the optimal *p* for average QFI and the optimal p for average fidelity both still need numerical calculation to decide.

#### Weak measurement and quantum measurement reversal

For comparing QFI with fidelity, we also investigate another scheme which is called weak measurement and quantum measurement reversal (WMQMR)^17, 18, 51, 52^. WMQMR scheme has an advantage in protecting the fidelity, so we want to calculate the average QFI and average fidelity and to see if the two protections are consistent with each other. Let us introduce this scheme. At first, the scheme is to use WM on each of the *N*-qubit generalized GHZ state before ADC. And then the *N*-qubit generalized GHZ state pass through the ADC. After the ADC, we use reversing measurement on each of the *N* qubits, Hence we can get the evolved *ρ* ^*out*^. This is the overall process of WMQMR. The WM operator of WMQMR is $${M}^{wm}={(\begin{array}{cc}1 & 0\\ 0 & \sqrt{1-{p}_{1h}}\end{array})}^{\otimes N}$$ and reversing measurement operator of WMQMR is $${M}^{rev}={(\begin{array}{cc}\sqrt{1-{p}_{rh}} & 0\\ 0 & 1\end{array})}^{\otimes N}$$, $(h=1,2,3,\ldots ,N)$. As the weak measurement and quantum measurement reversal only have one measurement operator respectively on each qubit, they are incomplete (or partial) measurements on *N*-qubit. Hence they only have one successful case while the other cases have been discarded. And here the two WMs do not be accompanied with the pre- and post-flips before and after the ADC respectively.

Based on the Additional information, we can calculate the average QFI of the WMQMR

4 $$\begin{array}{rcl}{F}_{WMQMR} & = & 4{|C|}^{2}{N}^{2}/(A+B)\\  & = & \tfrac{4{|\alpha \beta |}^{2}{\prod }_{h}\mathrm{(1}-{r}_{h}){N}^{2}}{\tfrac{{|\alpha |}^{2}}{{\prod }_{h}\mathrm{(1}-{p}_{1h})}+{|\beta |}^{2}(\tfrac{{\prod }_{h}{s}_{h}}{{\prod }_{h}\mathrm{(1}-{p}_{rh})}+{\prod }_{h}{r}_{h})}\\  & \mathop{=}\limits^{S} & \tfrac{4{|\alpha \beta |}^{2}{\mathrm{(1}-r)}^{N}{N}^{2}}{\tfrac{{|\alpha |}^{2}}{{\mathrm{(1}-{p}_{1})}^{N}}+{|\beta |}^{2}(\tfrac{{\mathrm{(1}-r)}^{N}}{{\mathrm{(1}-{p}_{r})}^{N}}+{r}^{N})},\end{array}$$

and the probability and the fidelity of it with the same parameters and same ADCs. The success probability of the WMQMR is

5 $$\begin{array}{rcl}{P}_{WMQMR} & = & {|\alpha |}^{2}\prod _{h}(1-{p}_{rh})+{|\beta |}^{2}\prod _{h}\mathrm{(1}-{p}_{1h})({r}_{h}\mathrm{(1}-{p}_{rh})+{s}_{h})\\  & \mathop{=}\limits^{S} & {|\alpha |}^{2}{\mathrm{(1}-{p}_{r})}^{N}+{|\beta |}^{2}{\mathrm{(1}-{p}_{1})}^{N}{(r(1-{p}_{r})+s)}^{N},\end{array}$$

and average fidelity of the WMQMR is

6 $$\begin{array}{ccc}Fi{d}_{WMQMR} & = & \frac{1}{{P}_{WMQMR}}[{|\alpha |}^{4}\prod _{h}{(1-{p}_{rh})}^{N}+{|\alpha \beta |}^{2}\prod _{h}(1-{p}_{1h}){r}_{h}(1-{p}_{rh})\\  &  & +2{|\alpha \beta |}^{2}\prod _{h}{(1-{p}_{1h})}^{\frac{1}{2}}{s}_{h}^{\frac{1}{2}}{(1-{p}_{rh})}^{\frac{1}{2}}+{|\beta |}^{4}\prod _{h}(1-{p}_{1h}){s}_{h}]\\  & \mathop{=}\limits^{S} & \frac{1}{{P}_{WMQMR}}[{|\alpha |}^{4}{(1-{p}_{r})}^{N}+{|\alpha \beta |}^{2}{(1-{p}_{1})}^{N}{r}^{N}{(1-{p}_{r})}^{N}\\  &  & +2{|\alpha \beta |}^{2}{(1-{p}_{1})}^{\frac{N}{2}}{s}^{\frac{N}{2}}{(1-{p}_{r})}^{\frac{N}{2}}+{|\beta |}^{4}{(1-{p}_{1})}^{N}{s}^{N}].\end{array}$$

Note that here the average fidelity *Fid*_*WMQMR*_ has been divided by *P*_*WMQMR*_.

#### Do-nothing case

Substituting *p*_1*h*_ = *p*_*rh*_ = 0 $(h=1,2\ldots N)$ into Eqs (4), (5) and (6) for *WMQMR* case, we can get the average QFI, probability and average fidelity of *N*-qubit generalized GHZ state for general versions and simplified versions of the DN case (i.e., pure evolution of ADC), respectively.

7 $$\begin{array}{rcl}{F}_{DN} & = & 4{|\alpha \beta |}^{2}\prod _{h}(1-{r}_{h}){N}^{2}/({|\alpha |}^{2}+{|\beta |}^{2}(\prod _{h}(1-{r}_{h})+\prod _{h}{r}_{h}))\\  & \mathop{=}\limits^{S} & 4{|\alpha \beta |}^{2}{\mathrm{(1}-r)}^{N}{N}^{2}/({|\alpha |}^{2}+{|\beta |}^{2}\mathrm{((1}-r{)}^{N}+{r}^{N})),\end{array}$$

and the probability of the DN case is ${P}_{DN}\equiv 1$ for general version and simplified version. And average fidelity of the DN case is

8 $$\begin{array}{rcl}Fi{d}_{DN} & = & \frac{1}{{P}_{DN}}[{|\alpha |}^{4}+{|\alpha \beta |}^{2}\prod _{h}{r}_{h}+2{|\alpha \beta |}^{2}\prod _{h}{s}_{h}^{\frac{1}{2}}+{|\beta |}^{4}\prod _{h}{s}_{h}]\\  & \mathop{=}\limits^{S} & [{|\alpha |}^{4}+{|\alpha \beta |}^{2}{r}^{N}+2{|\alpha \beta |}^{2}{s}^{\frac{N}{2}}+{|\beta |}^{4}{s}^{N}].\end{array}$$

Comparing Eq. (7) with Eq. (4), we can find that *F*_*DN*_ is the maximum of *F*_*WMQMR*_ when *p*_1*h*_ = *p*_*rh*_ = 0 $(h=1,2\ldots N)$, which means WMQMR scheme in average QFI is worthless to discuss. However, its average fidelity is higher than the *Fid*_*DN*_ and so worth discussing.

### Analysis

**Figure 2:**
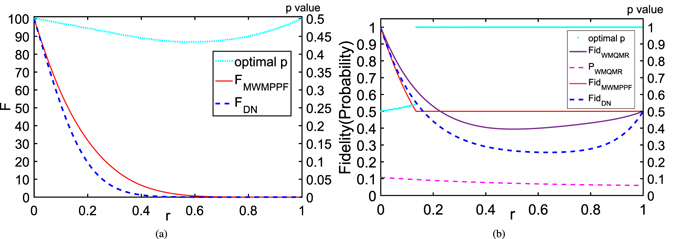
(**a**) Average QFI and its optimal *p* vs. *r* with *N* = 10, *θ* = *π*/2. (1) *F*_*MWMPPF*_ (red full line) is the maximal average QFI of WMPPF: *F*_*WMPPF*_ by choosing the optimal *p* value as *r* varies; (2) the optimal *p* value (celeste dotted line) for *F*_*MWMPPF*_ according to *r* can be drawn at the same parameters with the coordinate scale on the right edge of the figure; (3) *F*_*DN*_ (blue dashed line) for DN case. (**b**) Average fidelity and its optimal *p* and probability vs. *r* with *N* = 10, *θ* = *π*/2. (1) *Fid*_*MWMPPF*_ (asterisk line): the maximal average fidelity of WMPPF; (2) the optimal *p* value (celeste dotted line) for *Fid*_*MWMPPF*_ can be drawn at the same parameters with the coordinate scale on the right edge of the figure; (3) *Fid*_*MWQMR*_ (purple full line), we provide its *p*_1_ = *p*_*r*_ = 0.2 as an example and its probability is: (4) *P*_*WMQMR*_ (magenta dashed line) with the same parameters; (5) *Fid*_*DN*_ (blue dashed line).

We now proceed to analyze the figures we have drawn. For simplicity, all the figures we will discuss are based on the simplified version formulas mentioned before. In this paper, WMPPF is the scheme whose WM strength *p* is not optimized for QFI or fidelity, and *p* can be any value in $[0,1]$ except being provided beforehand. And MWMPPF indicates the maximal average QFI or average fidelity of WMPPF by the optimization of some parameters, e.g., *p*. It indicate maximal protection of WMPPF on QFI or fidelity. In this paper we use WMPPF to indicate not optimized scheme and MWMPPF to indicate optimized one. below we sometimes use WMPPF and sometimes use MWMPPF which collectively refers to WMPPF scheme. Figure 2(a) is the average QFI vs. *r* with *N* = 10, *θ* = *π*/2. It is easy to find from the figure that the MWMPPF scheme is all higher than the DN case, which means by using the MWMPPF we can improve the measurement accuracy of the phase. *F*_*MWMPPF*_ is the maximum of the *F*_*WMPPF*_ by adjusting the parameter *p*, which is always 0.5 at *r* = 0 but it may slightly deviate from 0.5 when *r* > 0, and this is demonstrated as celeste dot line in Fig. 2(a). In WMPPF, we only use the WM but do not use the reversing measurement^51^ because the non-complete reversing measurement can decrease the probability and hence can heavily decrease the average QFI. The WMQMR get lower average QFI than the DN case also because the reversing measurement will decrease the probability and so greatly decreases the average QFI. Here note that in Fig. 2(a) we do not draw the curve of the average QFI of GHZ state for WMQMR because it is lower than *F*_*DN*_ for all *r* in $[0,1]$, which has been mentioned already. Figure 2(b) shows average fidelity vs. *r* for the same situation with Fig. 2(a). From it, *Fid*_*MWMPPF*_ (we call *Fid*_*MWMPPF*_ the maximal average fidelity of *Fid*_*WMPPF*_, which depends on different optimized *p* on different *r*) are not always larger than *Fid*_*DN*_. For *Fid*_*WMQMR*_, we provide *p*_1_ = *p*_*r*_ = 0.2 as an example. Contrasting (a) with (b) on the same *r* infers that: the WMPPF can protect the average QFI but this scheme can not protect fidelity very well even for MWMPPF, and WMQMR can protect the average fidelity but can not protect the average QFI at all. So for WMPPF or for WMQMR, a scheme protecting the average fidelity of GHZ state does not necessarily protect the average QFI of it and vice versa.

**Figure 3:**
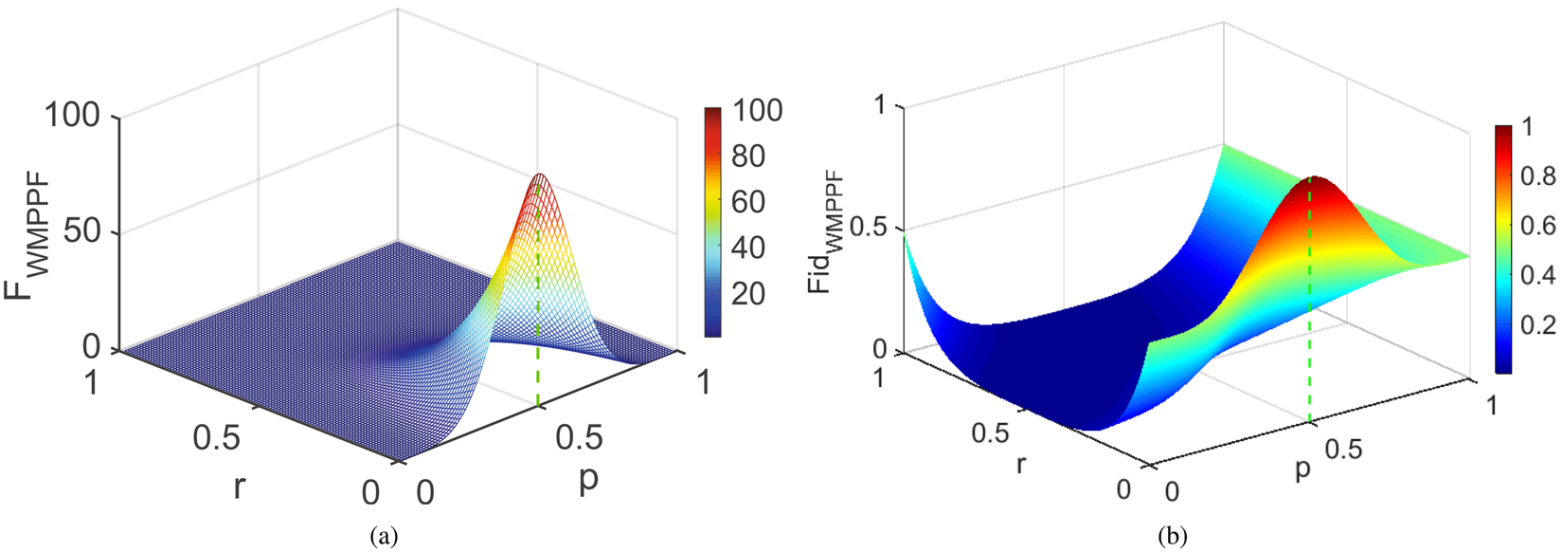
(**a**) Average QFI vs. *p* and *r* for WMPPF with *N* = 10, *θ* = *π*/2. (**b**) Average fidelity vs. *p* and r for WMPPF with *N* = 10, *θ* = *π*/2. In (**a**,**b**), the maximal values of *F*_*WMPPF*_ (i.e., the maximal average QFI: *F*_*MWMPPF*_) and *Fid*_*WMPPF*_ (i.e., the maximal average fidelity: *Fid*_*MWMPPF*_) are corresponding to the ridge lines of the two figures, respectively. It is clear that *p* = 0.5 is the optimal value for *F*_*MWMPPF*_ and *Fid*_*MWMPPF*_ at *r* = 0, while the optimal *p* values for *F*_*MWMPPF*_ and for *Fid*_*MWMPPF*_ are deviated from 0.5 with different features when *r* > 0.

In Fig. 3(a) we give the average QFI of WMPPF vs. *p* and *r* with *N* = 10, *θ* = *π*/2. In Fig. 3(b), we give the average fidelity of WMPPF vs. *p* and *r* with the same parameters as Fig. 3(a). From them we can see that *F*_*MWMPPF*_ and *Fid*_*MWMPPF*_ depends different optimized *p* with different *r* > 0, which are the maximal average QFI of the WMPPF *F*_*WMPPF*_ and the maximal average fidelity of the WMPPF *Fid*_*WMPPF*_ corresponding to the ridge lines of the two figures respectively. And the two *p*-*r*-plane projection lines of these two ridge lines are just the *p* value curves in Fig. 2(a,b) respectively. It is clear that *p* = 0.5 is the optimal value for both *F*_*MWMPPF*_ and *Fid*_*MWMPPF*_ at *r* = 0 (i.e., 100 and 1 separately), while the optimal *p* values for *F*_*MWMPPF*_ and for *Fid*_*MWMPPF*_ are different and they both deviated from 0.5 when *r* > 0. And the two figures depict that their maximal values evolving with *r* > 0 are very different, respectively. In Fig. 3(b) when *r* changes to 0.134 where the optimal *p* of the average fidelity is 0.538 and the maximal average fidelity is 0.5005. However, when *r* = 0.135 or larger than it, the maximal average fidelity *Fid*_*MWMPPF*_ will become 0.5 with *p* = 1 and the measurement style suddenly jumps to strong measurement. This means strong measurement will has the advantage than the WM after this turning point. This also gives the reason why *Fid*_*MWMPPF*_ in Fig. 2(b) has a transition by a turning point. Contrasting Fig. 3(a) with (b) infers that even on the same *r* > 0 and *p* for WMPPF, the evolving of the maximal average QFI and the maximal average fidelity are very different, i.e., protecting the average fidelity does not necessarily protect the average QFI and vice versa.

**Figure 4:**
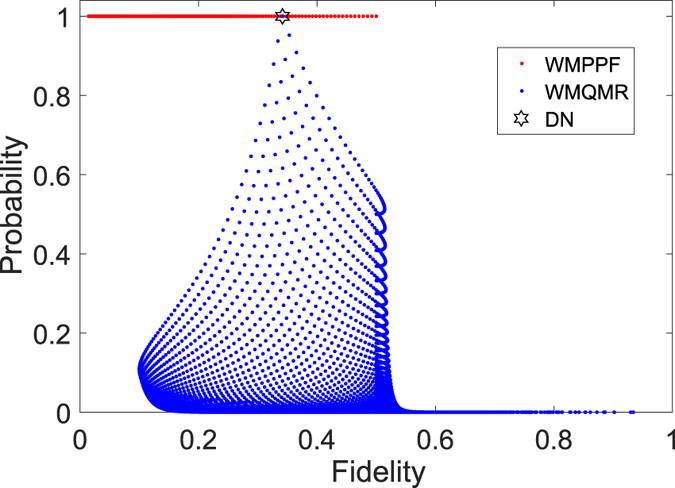
Average fidelity vs. probability with *N* = 10, *θ* = *π*/2, *r* = 0.3. (1) *Fid*_*WMPPF*_ vs. *P*_*WMPPF*_ with red dot, the top red line indicate the probability 1. (2) *Fid*_*WMQMR*_ vs. *P*_*WMQMR*_ with blue dot while *p*_1_ and *p*_*r*_ can be any value of the range $[0,1]$. (3) *Fid*_*DN*_ in this condition only has a value and *P*_*DN*_ = 1, which we plot a hexagram to indicate. And for WMQMR, the top blue point in the heart of the hexagram, which indicates that the top blue point is actually the DN case (i.e., *p*_1_ = *p*_*r*_ = 0) and its probability is 1. WMPPF can get better average fidelity than WMQMR when WMQMR has large probability (i.e., near 1).

In Fig. 4, we can see WMPPF can get better average fidelity than WMQMR when WMQMR has large probability (i.e., *P*_*WMQMR*_ is toward 1) and *r* is not too small. And if we choose *p*_1_ = *p*_*r*_→1 (i.e., toward strong measurement) the probability *P*_*WMQMR*_ is so small that is toward 0, the average fidelity *Fid*_*WMQMR*_ is toward 1. This is consistent with the results of refs 17, 51 and 53 whose core idea is greatly increasing the fidelity or concurrence at the cost of greatly decreasing the probability.

**Figure 5:**
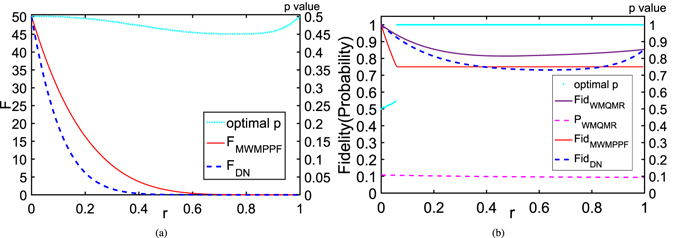
(**a**) Average QFI and the optimal *p* vs. *r* with *N* = 10, *θ* = *π*/4. (1) *F*_*MWMPPF*_ (red full line) is the maximum of *F*_*WMPPF*_ by choosing the optimal *p* value as *r* varies; (2) the optimal *p* value for average QFI of *F*_*MWMPPF*_ (celeste dotted line) according to *r* can be drawn at the same parameters with the coordinate scale on the right edge of the figure; (3) *F*_*DN*_ (blue dashed line) for DN case. (**b**) Average fidelity and the optimal *p* and probability vs. *r* with *N* = 10, *θ* = *π*/4. (1) *Fid*_*WMQMR*_ (purple full line) is the average fidelity of WMQMR, we provide its *p*_1_ = *p*_*r*_ = 0.2 as an example and its probability is: (2) *P*_*WMQMR*_ (magenta dashed line) with the same parameters; (3) *Fid*_*MWMPPF*_ (asterisk line); (4) the optimal *p* value for *Fid*_*MWMPPF*_ (celeste dotted line) as *r* varies; (5) *Fid*_*DN*_ (blue dashed line).

Above we mainly discuss the *N*-qubit GHZ state where |*α*| = |*β*| (*θ* = *π*/2) and *ϕ*_0_ = 0. If $|\alpha |\ne |\beta |$ $(\theta \ne \pi /2)$, it is non-maximally entangled state or generalized GHZ state. Numerical calculations show that when *α* > *β* (0 < *θ* < *π*/2), the WMPPF can protect the generalized GHZ state in average QFI like GHZ state discussed while it can not protect the average fidelity well. Here we draw Fig. 5 to show this case as an example. Figure 5(a) shows that the WMPPF can protect the average QFI of the generalized GHZ state (e.g., *θ* = *π*/4) just as GHZ state. Figure 5(b) shows that WMQMR can give better protection for the generalized GHZ state (*θ* = *π*/4) on average fidelity than WMPPF while its probability does not decrease too much, and it is obviously that the MWMPPF has nearly less average fidelity than DN case when *r* is not too large and too small. Although WMPPF in this case does not protect well the state which initially is GHZ state, it can protect the average QFI and can acquire the high measurement precision of the phase. This gives us a conclusion that when the state evolving with *r* from its initial GHZ state in ADC, maybe our WMPPF scheme causes larger deviation than the DN case while phase measurement of the former get larger precision than the latter.

Both Figs 2 and 5 display that the optimal *p* values for average QFIs that are plotted with celeste dotted lines are not in accordance with the optimal *p* value for average fidelities. It can be inferred that the optimizing average QFI and average fidelity by *p* are two different things that need not be consistent. Sometimes they may be conflict to each other. Contrasting (a) with (b) of both Figs 2 and 5 on the same *r* infers that: the WMPPF can protect the average QFI but this scheme can not protect fidelity very well even for MWMPPF, and WMQMR can protect the average fidelity but can not protect the average QFI at all (note that we do not draw the *F*_*WMQMR*_ in Figs 2 and 5). So for WMPPF or for WMQMR, a scheme protecting the average fidelity of GHZ state does not necessarily protect the average QFI of it and vice versa. Hence, if we want to seek the way of protecting average QFI, protecting the state is not always effective. The optimal *p* for average QFI demonstrated in Figs 2 and 5 are around to 0.5. *p* = 0.5 means the weak measurement and the pre-flips can be replaced by stochastic $\tfrac{1}{2}$-probability pre-flips for all qubits of the GHZ state or for some generalized GHZ state, where post-flip flipping or not is still based on the pre-flip flipping or not for each qubit. And if needed, *p* = 0.5 can be used for implementing the approximated MWMPPF for average-QFI protection with simpler process and less apparatus, and the average QFI can decrease not too much comparing to the strict MWMPPF which comes from optimized WMPPF by *p*.

**Figure 6:**
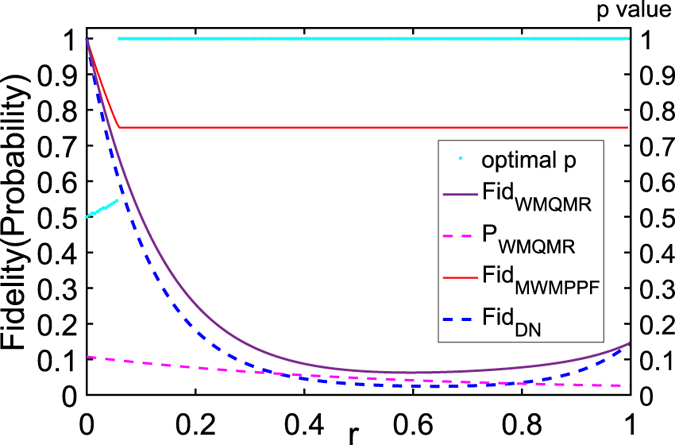
Average fidelity and probability and the optimal *p* value vs. *r* with *θ* = 3*π*/4. (1) *Fid*_*DN*_ (blue dashed line); (2) *Fid*_*MWMPPF*_ (red full line) under its optimal *p* value evolves with *r*; (3) the optimal *p* value of average fidelity (celeste dotted line) evolves with *r*; (4) *Fid*_*WMQMR*_ (purple full line) with its *p*_1_ = *p*_*r*_ = 0.2 and its probability is: (5) *P*_*WMQMR*_ (magenta dashed line) with the same parameters.

When *π*/2 < *θ* < *π* (|*α*| < |*β*|), average-QFI protection of WMPPF can be effective only when *r* is in the range close to 1 due to $[0,1]$, and for simplicity here we do not draw the figure to display it. Actually, when *θ*→*π* $(|\alpha |\ll |\beta |)$, the WMPPF can do much more protecting of the average fidelity of this generalized GHZ state than WMQMR does (e.g., *θ* = 3*π*/4 in Fig. 6). In Fig. 6, we provide *p*_1_ = *p*_*r*_ = 0.2 to WMQMR as an example. However, in this case, average-QFI protection can be effective only when *r* is in a very narrow range of $[0,1]$ that is close to 1. So in this case, it needs a trade off between the WMPPF and DN.

It is clear from above figures of fidelity in this paper that different schemes mentioned have different advantages for different *r*. So if in practise, we need a trade off to use the different schemes or even DN case to optimize the average fidelity for different *r*.

**Figure 7:**
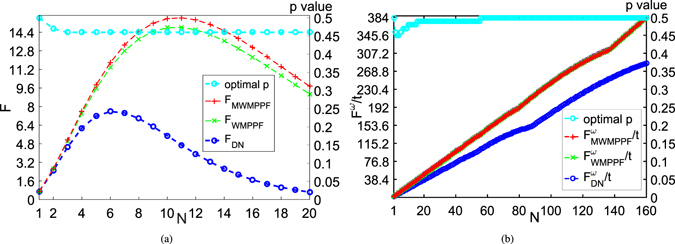
(**a**) Average QFI of phase *ϕ* vs. *N* and optimal *p* of *F*_*MWMPPF*_ vs. *N* with *N* = 1 to 20 while *r* = 0.3, *θ* = *π*/2. (1) *F*_*DN*_ (blue dashed line) for DN case; (2) *F*_*WMPPF*_ (green dashed line), provided that *p* is fixed with 0.5; (3) *F*_*WMWPPF*_ (red dashed line) is the maximum of *F*_*WMPPF*_ by choosing the optimal *p* value when *r* = 0.3; (4) the optimal *p* value for average QFI (celeste dashed line) according to *N* can be drawn at the same parameters with the coordinate scale on the right edge of the figure, which is the *p* of *F*_*MWMPPF*_. (**b**) Average QFI of frequency *ω* divided by *t* (or average QFI of phase multiplying *t*) vs. *N* and optimal *p* of ${F}_{MWMPPF}^{\omega }/t$ vs. *N* with *N* = 1 to 160 and Γ = 0.3 while *t* and *p* are both optimized. (1) ${F}_{DN}^{\omega }/t$ (blue dashed line) for DN case; (2) ${F}_{WMPPF}^{\omega }/t$ (green dashed line), provided that *p* is fixed with 0.5; (3) ${F}_{MWMPPF}^{\omega }/t$ (red dashed line) is the maximum of ${F}_{WMPPF}^{\omega }/t$ by choosing the optimal *p* value when *r* = 0.3, which is very close to ${F}_{WMPPF}^{\omega }/t$ especially when *N* ≥ 56; (4) the optimal *p* value for average QFI (celeste dashed line) according to *N* can be drawn at the same parameters with the coordinate scale on the right edge of the figure.

In Fig. 7(a), we depict the QFI of phase estimation against qubit number *N*, i.e., average QFI of phase *ϕ* and the optimal *p* vs. *N* with *N* = 1 to 20 while *r* = 0.3. From it, the *F*_*WMPPF*_ is larger than *F*_*DN*_ and the *F*_*MWMPPF*_ is the maximum of *F*_*WMPPF*_ by optimize the value of *p*, and the optimal *N* of *F*_*MWMPPF*_, *F*_*WMPPF*_ and *F*_*DN*_ are 11, 11 and 6, respectively. Moreover, numerical calculation shows that if *r* is smaller the optimal *N* for the three ones will become larger, e.g., if we we only change *r* to 0.1 and keep other parameters used in Fig. 7(a) unchanged, the optimal *N* of the three ones are 37, 37 and 21. Now we consider to the frequency estimation. We know that here the phase is accumulated by the frequency with time instead of the phase gate of phase estimation. As the estimated parameter now corresponds to *ω* = *ϕ*/*t* where *ϕ* is the phase of the unitary rotation by the phase gate, the QFI via a parameter change just rescales. This change can be true for our phase gate ${U}_{\varphi }={e}^{-i{\sigma }_{z}\varphi \mathrm{/2}}$ is commutate with the WMPPF. In frequency estimation, *F* ^*ω*^/*t* is usually the objective we are focused. Here Fig. 7(b) is about average QFI of frequency *ω* divided by *t* and *p* of *F*_*MWMPPF*_ (or the optimal *p* of *F*_*WMPPF*_) vs. *N* with *N* = 1 to 160 and Γ = 0.3 while *p* and *t* are optimized. Note here in Fig. 7(a) only *p* is optimized. As average QFI of frequency is the average QFI of phase multiplying ${t}^{2}:{F}^{\omega }=F\ast {t}^{2}$, so in Fig. 7(b) average QFI of *ω* divided by *t* can be expressed by average QFI of phase multiplying *t*: *F* ^*ω*^/*t* = *Ft* ^10, 12, 32^. This then give us a way to directly calculate the average QFI of frequency by the former formulas of calculating the average QFI of phase. So in this situation, we can equally express *F* ^*ω*^/*t* as *Ft* for different cases(i.e., WMPPF, DN). Note that here we still need not to consider WMQMR case for it is always smaller than DN according to the calculations. Thus from Fig. 7(b), it is easy to see that the *F*_*WMPPF*_ *t* is larger than *F*_*DN*_ *t*. And *F*_*MWMPPF*_ *t* is nearly coincident with the *F*_*WMPPF*_ *t* with *p* = 0.5. Especially when *N* ≥ 56, they are completely the same curve, which means in this case (i.e., *N* ≥ 56) the optimal *p* value is equal to 0.5. In Fig. 7, comparing MWMPPF with WMPPF whose *p* is 0.5, we can find that WM make more contribution in protecting the average QFIs of phase than that of frequency. Comparing with the DN scheme according to both *N* and the optimal *N* (i.e., *N* according to peak value of QFI) in Fig. 7(a,b), we can see that WMPPF scheme can get higher average QFIs, which means it is effective in protecting the average QFIs on phase estimation and frequency estimation.

**Figure 8:**
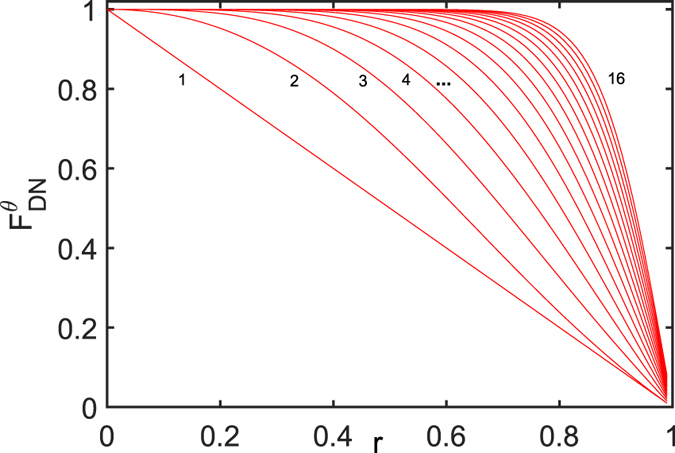
${F}_{DN}^{\theta }$ vs. *r* for GHZ state with *θ*  = *π*/2 and $N=1,2,3,4\ldots 16$ (corresponding to the red full line from the left to the right separately as tagged in the figure). It is clear from it that the lager *N* leads to lager QFI for any *r*.

Below we turn to discuss the QFI of weight factor *θ* of the initial state for the three cases, where the initial state has been given earlier in this article. From the detailed analysis in the Additional information, we know that WMPPF can only protect the average QFI of weight factor *θ* when *θ* > *π/2* and *r* is not small (especially, when *θ* is closer to *π*, *r* can be smaller). We draw a figure in the Additional information to show it. Calculations show that the QFI of *θ* of WMQMR is always lower than that of DN for any ${p}_{1h}\ne 0$, ${p}_{rh}\ne 0$ and *r*_*h*_ $(h=1\ldots N)$ to any *θ* and any qubit number *N*, so we need not to discuss it. Comparing the WMPPF and DN, when *θ* ≤ *π/2﻿,* or *θ* > *π/2* but *r* is small, DN has advantage. And based on many numeric calculations, for any *θ* and any *r*, when *N* increases, both QFIs for WMPPF and DN will increase. Here for simplicity, we only draw a figure (i.e., Fig. 8) of QFI of DN to show this character. In Fig. 8, when *N* is changed from 1 to 16 for GHZ state (thus *θ* = *π*/2), QFIs in the curves gradually increase corresponding from the left to the right as tagged in the figure (Note that here when *N* = 1, ${F}_{DN}^{\theta }$ is $s\equiv 1-r$ which is just the same expression as in the work^36^). So we can give a conclusion that increasing the qubit number N of GHZ state or generalized GHZ state will help us to resist the dissipation of ADC.

## Discussion

In this paper, for resist the dissipation of the QFI, we investigate the effect of the protocol by using the tools: WM, pre-flips and post-flips, and give the general formulas to calculate the average QFIs and average fidelities for both GHZ state and generalized GHZ states. Based on the formulas we have demonstrated that we can effectively protect average QFIs of the phase and frequency of the *N*-qubit GHZ state and some generalized GHZ state in ADC for any 0 < *θ* ≤ *π*/2, any *ϕ* and *ϕ*_0_, which is our main result in this paper. And when *π*/2 < *θ* < *π*, the average-QFI protection is effective only for *r* near 1. However, the WMQMR scheme is not useful in average QFI in ADC for GHZ state and generalized GHZ states. Our scheme is different from the former reaserch^51^ which has used reversing measurement However, the reversing measurement will decrease the probability for its non-complete measurement. This will bring us a question whether we will count the failed measurements result into the total measurement times. For fairly comparison, we count them into the total measurement times, and numerical calculations infer that the decreasing of the probability is harmful to the average QFI. Therefore, we do not use reversing measurement in our WMPPF scheme. We have also displayed that the evolving average fidelity of WMPPF, WMQMR and the DN case, which shows that the WMPPF scheme has some advantages on the average fidelity than the DN case for *N*-qubit GHZ state only at some special time. And the WMQMR scheme have advantages on both GHZ state and generalized GHZ states, with the cost of a decreasing probability. So for fidelity protection, it needs a trade off between the success probability and the average fidelity for WMQMR. In this paper, contrasting QFI with fidelity on the same *r* infers that, for WMPPF or for WMQMR, a scheme protecting the average fidelity of GHZ state does not necessarily protect the average QFI of it and vice versa, and this is our another result. Finally, WMPPF scheme is superior than DN scheme according to both *N* and the optimal *N* in phase estimation or frequency estimation. This can give us a reference to choose the optimal *N* for phase or frequency measurement according to our scheme WMPPF. As to the QFI of the weight factor investigated, our scheme can only protect some of the generalized GHZ state and for some special time. Comparing our scheme with the DN case, we also find that for pure ADC (i.e.,DN case), QFI of weight factor can be protected by increasing the qubit number *N*, and this conclusion may be valuable for the quantum metrology. It is worth noting that the WM can be easily applied to any types of qubit such as optical polarization qubits, particle-spin qubits^54, 55^ and Josephson junction qubits, etc. The experimental implementation of WM was realized recently in a photonic architecture^17, 19, 26, 27^, and the WM has also been demonstrated experimentally with Josephson junction^20^. Therefore, our scheme is a simple and direct method that is entirely feasible with current technology and available not only for optical instruments but also for atomic ones according to the GHZ state and generalized GHZ state. We hope that our scheme can be used in the future.

## Methods

### The general formula of QFI of phase to the output matrix with special structure

Here we will derive the general formula used for the calculation of average QFI of phase *ϕ* on both WMPPF and WMQMR. In this paper, we can apply this general formula to calculate their average QFIs since their matrices of *ρ* ^*out*^ have the same structure:

9 $${\rho }^{out}=\frac{1}{P}\tilde{\rho }=\frac{1}{P}(\begin{array}{ccc}A & {\bf{0}} & D\\ {\bf{0}} & E & {\bf{0}}\\ C & {\bf{0}} & B\end{array}).$$

$P=Tr[\tilde{\rho }]=A+B+Tr(E)$, which means the appearing probability of *ρ* ^*out*^, is the trace of $\tilde{\rho }$. Here $\tilde{\rho }$ is the unnormalized output matrix. In the detailed derivation of the Additional information, We find that, for WMPPF case, *ρ* ^*out*^ to any of the 2^*N*^ WM results has this structure, and *ρ* ^*out*^ of WMQMR also has this structure although with only one WM result. So below we apply the general QFI formula to calculate the QFI of the output state *ρ* ^*out*^ with this structure which is suitable for both Eq. (9) and Eq. (22) of Additional information ^3, 34, 36, 56^:

10 $$\begin{array}{l}{F}_{\varphi }=\sum _{l^{\prime} }\frac{{({\partial }_{\varphi }{\lambda }_{l^{\prime} })}^{2}}{{\lambda }_{l^{\prime} }}+\sum _{l\ne m}\frac{\mathrm{2(}{\lambda }_{l}-{\lambda }_{m}{)}^{2}}{{\lambda }_{l}+{\lambda }_{m}}{|\langle {\phi }_{l}|{\partial }_{\varphi }|{\phi }_{m}\rangle |}^{2}.\end{array}$$

Here |*φ*_*m*_〉 (or |*φ*_*l*_〉) are eigenvectors of *ρ* ^*out*^. As *E* is independant of *ϕ*, the eigenvectors and eigenvalues of *E* have no contributions to the calculation of *F*_*ϕ*_, we need not to consider *E*. and only consider those of *ρ*_1_ whose bases are orthogonal to the bases of *E*. The eigenvalues of *ρ*_1_ of Eq. (10) (or Eq. (24)) in Additional information are:

11 $$\begin{array}{ccc}{\lambda }_{1} & = & [(A+B)+\sqrt{{(A+B)}^{2}-4(A\times B-C\times D)}]/(2P),\\ {\lambda }_{2} & = & [(A+B)-\sqrt{{(A+B)}^{2}-4(A\times B-C\times D)}]/(2P).\end{array}$$

And

12a $${\lambda }_{1}+{\lambda }_{2}=\frac{A}{P}+\frac{B}{P},$$

12b $${\lambda }_{1}{\lambda }_{2}=\frac{AB}{{P}^{2}}-\frac{CD}{{P}^{2}}.$$

And without loss of generality, the corresponding eigenvectors of *λ*_1_ and *λ*_2_ are assumed separately^49^:

13 $$\begin{array}{rcl}|{\phi }_{1}\rangle  & = & \cos \,\eta {|0\rangle }^{\otimes N}+\exp (i{\xi }_{1})\,\sin \,\eta {|1\rangle }^{\otimes N},\\ |{\phi }_{2}\rangle  & = & -\,\sin \,\eta {|0\rangle }^{\otimes N}+\exp (i{\xi }_{2})\,\cos \,\eta {|1\rangle }^{\otimes N}.\end{array}$$

We know that $C=|C|\,\exp (iN\varphi )$ and $D=|D|\,\exp (-iN\varphi )$, where |*C*| = |*D*|. By the calculation of the eigenvalue equation, we can get ${\xi }_{1}={\xi }_{2}=N\varphi$, and the two eigenvalues ${\lambda }_{1}=(A+|D|\,\tan \,\eta )/P=(B+|C|\,\cot \,\eta )/P$, ${\lambda }_{2}=(B-|C|\,\tan \,\eta )/P=(A-|D|\,\cot \,\eta )/P$. So substituting *λ*_1_ of Eq. (11) into the above *λ*_1_, we have $\eta \,=$ $\arctan (\frac{-(A-B)+\sqrt{{(A-B)}^{2}+4C\times D}}{2|C|}) > 0$. So we know that our assumption of eigenvectors infers $\eta \,=$ $\arctan (\frac{-(A-B)+\sqrt{{(A-B)}^{2}+4C\times D}}{\mathrm{2|}C|})\in (0,\pi \mathrm{/2})$. And *λ*_1_ multiplied by *λ*_2_ gives

14 $${\lambda }_{1}{\lambda }_{2}=\frac{AB+(B|D|-A|C|)\,\tan \,\eta -|C||D|\,{\tan }^{2}\,\eta }{{P}^{2}}.$$

From Eq. (14), Eq. (12b) and $\tan \,\mathrm{(2}\eta )=\frac{2\,\tan \,\eta }{1-{\tan }^{2}\,\eta }$, we can get

15 $$\tan \,\mathrm{(2}\eta )=\frac{2|C|}{A-B}.$$

As the eigenvalues and the eigenvectors of *ρ* ^*out*^ are independent with *ϕ* except |*φ*_1_〉 and |*φ*_2_〉 of *ρ*_1_, Eq. (10) can be simplified as

16 $$\begin{array}{rcl}{F}_{\varphi } & = & 4\frac{{({\lambda }_{1}-{\lambda }_{2})}^{2}}{{\lambda }_{1}+{\lambda }_{2}}{(\sin \eta \cos \eta )}^{2}{N}^{2}\\  & = & \frac{1}{P}\frac{\mathrm{4[(}A+B{)}^{2}-\mathrm{4(}AB-CD)]}{A+B}\frac{{\sin }^{2}\,\mathrm{(2}\eta )}{4}{N}^{2}\\  & = & \frac{1}{P}\frac{4{|C|}^{2}}{A+B}{N}^{2}.\end{array}$$

Here ${\sin }^{2}\mathrm{(2}\eta )=\frac{4{|C|}^{2}}{{(A+B)}^{2}-\mathrm{4(}AB-CD)}$ can be gotten from Eq. (15).

## Electronic supplementary material

Supplementary Information

## References

[1] Giovannetti V, Lloyd S, Maccone L (2004). Quantum-Enhanced Measurements: Beating the Standard Quantum Limit. Science.

[2] Dorner U (2012). Quantum frequency estimation with trapped ions and atoms. New Journal of Physics.

[3] Braunstein SL, Caves CM (1994). Statistical distance and the geometry of quantum states. Phys. Rev. Lett..

[4] Zheng Q, Yao Y, Li Y (2016). Optimal quantum parameter estimation in a pulsed quantum optomechanical system. Phys. Rev. A.

[5] Wang, Z. H., Zheng, Q., Wang, X. & Li, Y. The energy-level crossing behavior and quantum fisher information in a quantum well with spin-orbit coupling. *Scientific Reports* **6**, 22347 EP – (2016). 10.1038/srep22347 PMC4773991 26931762

[6] Jin, Y. The effects of vacuum fluctuations on teleportation of quantum fisher information. *Scientific Reports* **7**, 40193 EP – (2017). 10.1038/srep40193 PMC5220366 28067291

[7] Sekatski P, Skotiniotis M, Dür W (2016). Dynamical decoupling leads to improved scaling in noisy quantum metrology. New Journal of Physics.

[8] Tan Q-S, Huang Y, Yin X, Kuang L-M, Wang X (2013). Enhancement of parameter-estimation precision in noisy systems by dynamical decoupling pulses. Phys. Rev. A.

[9] Kominis IK (2008). Sub-Shot-Noise Magnetometry with a Correlated Spin-Relaxation Dominated Alkali-Metal Vapor. Phys. Rev. Lett..

[10] Dür W, Skotiniotis M, Fröwis F, Kraus B (2014). Improved Quantum Metrology Using Quantum Error Correction. Phys. Rev. Lett..

[11] Kessler EM, Lovchinsky I, Sushkov AO, Lukin MD (2014). Quantum Error Correction for Metrology. Phys. Rev. Lett..

[12] Kołodyński J, Demkowicz-Dobrzański R (2013). Efficient tools for quantum metrology with uncorrelated noise. New Journal of Physics.

[13] Demkowicz-Dobrzański R, Maccone L (2014). Using Entanglement Against Noise in Quantum Metrology. Phys. Rev. Lett..

[14] Huang Z, Macchiavello C, Maccone L (2016). Usefulness of entanglement-assisted quantum metrology. Phys. Rev. A.

[15] Wang Y-T (2016). Experimental Demonstration of Higher Precision Weak-Value-Based Metrology Using Power Recycling. Phys. Rev. Lett..

[16] Jordan AN, Martnez-Rincón J, Howell JC (2014). Technical Advantages for Weak-Value Amplification: When Less Is More. Phys. Rev. X.

[17] Kim Y-S, Lee J-C, Kwon O, Kim Y-H (2012). Protecting entanglement from decoherence using weak measurement and quantum measurement reversal. Nat Phys.

[18] Brańczyk AM, Mendonça PEMF, Gilchrist A, Doherty AC, Bartlett SD (2007). Quantum control of a single qubit. Phys. Rev. A.

[19] Gillett GG (2010). Experimental Feedback Control of Quantum Systems Using Weak Measurements. Phys. Rev. Lett..

[20] Katz N (2008). Reversal of the Weak Measurement of a Quantum State in a Superconducting Phase Qubit. Phys. Rev. Lett..

[21] Pang S, Alonso JRG, Brun TA, Jordan AN (2016). Protecting weak measurements against systematic errors. Phys. Rev. A.

[22] Zheng Q, Ge L, Yao Y, Zhi Q-J (2015). Enhancing parameter precision of optimal quantum estimation by direct quantum feedback. Phys. Rev. A.

[23] Higgins BL, Berry DW, Bartlett SD, Wiseman HM, Pryde GJ (2007). Entanglement-free Heisenberg-limited phase estimation. Nature.

[24] Hatridge M (2013). Quantum Back-Action of an Individual Variable-Strength Measurement. Science.

[25] Wiseman HM, Milburn GJ (1993). Quantum theory of optical feedback via homodyne detection. Phys. Rev. Lett..

[26] Kim Y-S, Cho Y-W, Ra Y-S, Kim Y-H (2009). Reversing the weak quantum measurement for a photonic qubit. Opt. Express.

[27] Lee J-C, Jeong Y-C, Kim Y-S, Kim Y-H (2011). Experimental demonstration of decoherence suppression via quantum measurement reversal. Opt. Express.

[28] Pan J-W, Zeilinger A (1998). Greenberger-horne-zeilinger-state analyzer. Phys. Rev. A.

[29] Pan J-W, Bouwmeester D, Daniell M, Weinfurter H, Zeilinger A (2000). Experimental test of quantum nonlocality in three-photon Greenberger-Horne-Zeilinger entanglement. Nature.

[30] Jin, X.-M. *et al*. Sequential Path Entanglement for Quantum Metrology. *Scientific Reports* **3**, 1779 EP – (2013).

[31] Escher BM, de Matos Filho RL, Davidovich L (2011). General framework for estimating the ultimate precision limit in noisy quantum-enhanced metrology. Nat Phys.

[32] Chaves R, Brask JB, Markiewicz M, Kołodyński J, Acín A (2013). Noisy Metrology beyond the Standard Quantum Limit. Phys. Rev. Lett..

[33] Nielsen, M. A. & Chuang, I. L. Quantum noise and quantum operations. In *Quantum Computation and Quantum Information*: *10th Anniversary Edition*, 353–398 (Cambridge University Press, Cambridge, 2010).

[34] Ma J, Wang X, Sun C, Nori F (2011). Quantum spin squeezing. Physics Reports.

[35] Pezzé L, Smerzi A, Khoury G, Hodelin JF, Bouwmeester D (2007). Phase Detection at the Quantum Limit with Multiphoton Mach-Zehnder Interferometry. Phys. Rev. Lett..

[36] Zhong W, Sun Z, Ma J, Wang X, Nori F (2013). Fisher information under decoherence in Bloch representation. Phys. Rev. A.

[37] Gross C, Zibold T, Nicklas E, Estève J, Oberthaler MK (2010). Nonlinear atom interferometer surpasses classical precision limit. Nature.

[38] Huelga SF (1997). Improvement of Frequency Standards with Quantum Entanglement. Phys. Rev. Lett..

[39] Pezzé L, Smerzi A (2009). Entanglement, nonlinear dynamics, and the heisenberg limit. Phys. Rev. Lett..

[40] Guo, L.-S., Xu, B.-M., Zou, J. & Shao, B. Magnetic field sensing subject to correlated noise with a ring spin chain. *Scientific Reports* **6**, 33254 EP – (2016). 10.1038/srep33254 PMC5020690 27623048

[41] Giovannetti V, Lloyd S, Maccone L (2006). Quantum Metrology. Phys. Rev. Lett..

[42] Guo L-S, Xu B-M, Zou J, Shao B (2015). Improved thermometry of low-temperature quantum systems by a ring-structure probe. Phys. Rev. A.

[43] Berry DW, Wiseman HM (2000). Optimal States and Almost Optimal Adaptive Measurements for Quantum Interferometry. Phys. Rev. Lett..

[44] Kok P (2007). Linear optical quantum computing with photonic qubits. Rev. Mod. Phys..

[45] Knill E, Laflamme R, Milburn GJ (2001). A scheme for efficient quantum computation with linear optics. Nature.

[46] Demkowicz-Dobrzański, R., Kołodyński, J. & Guţă, M. The elusive Heisenberg limit in quantum-enhanced metrology. *Nature Communications* **3**, 1063 EP – (2012). 10.1038/ncomms2067 PMC3658100 22990859

[47] Micadei K (2015). Coherent measurements in quantum metrology. New Journal of Physics.

[48] Lu X-M, Wang X, Sun CP (2010). Quantum Fisher information flow and non-Markovian processes of open systems. Phys. Rev. A.

[49] Ma J, Huang Y-X, Wang X, Sun CP (2011). Quantum Fisher information of the Greenberger-Horne-Zeilinger state in decoherence channels. Phys. Rev. A.

[50] Wiseman HM (1995). Adaptive Phase Measurements of Optical Modes: Going Beyond the Marginal *Q* Distribution. Phys. Rev. Lett..

[51] Wang C-Q (2014). Feed-forward control for quantum state protection against decoherence. Phys. Rev. A.

[52] He Z, Yao C, Zou J (2013). Robust state transfer in the quantum spin channel via weak measurement and quantum measurement reversal. Phys. Rev. A.

[53] Korotkov AN, Keane K (2010). Decoherence suppression by quantum measurement reversal. Phys. Rev. A.

[54] Bollinger JJ, Itano WM, Wineland DJ, Heinzen DJ (1996). Optimal frequency measurements with maximally correlated states. Phys. Rev. A.

[55] Dowling JP (1998). Correlated input-port, matter-wave interferometer: Quantum-noise limits to the atom-laser gyroscope. Phys. Rev. A.

[56] Genoni MG, Olivares S, Paris MGA (2011). Optical Phase Estimation in the Presence of Phase Diffusion. Phys. Rev. Lett..

