# Supplementary material for: Protecting quantum Fisher information of N-qubit GHZ state by weak measurement with flips against dissipation
Source: Sci Rep. 2017 Jul 21;7:6160. doi: 10.1038/s41598-017-04726-1 (PMC5522470; doi:10.1038/s41598-017-04726-1)
Supplement: Supplementary file 1 — Supplementary Information [file 41598_2017_4726_MOESM1_ESM.pdf]

Additional information: Protecting quantum Fisher information of N-qubit GHZ state by weak measurement with flips against dissipation

Yu Chen[1,2,*], Jian Zou[1], Zheng-wen Long[3], and Bin Shao[1]

[1]School of Physics, Beijing Institute of Technology, Beijing 100081, China
[2]School of Physics and Electronic science, Guizhou Normal College, Guiyang 550018, China
[3]College of Physics, Guizhou University, Guiyang 550025, China
[*]chenyuchn@163.com

ABSTRACT

This **Additional information** has four sections. The first one is about the details of **The calculations of the output ρ^{out}, average QFI of phase and average fidelity for WMPPF**. The second one is about **The calculations of the output ρ^{out}, average QFI of phase and average fidelity for WMQMR**. The third one is about **Average QFIs of weight factor for WMQMR and DN**. The fourth one is about **Average QFI of weight factor for WMPPF**.

Additional information

The calculations of the output ρ^{out}, average QFI of phase and average fidelity for WMPPF. We choose $M_1^i(p_i)$ and $M_2^j(p_j)$ to indicate the two WM solutions respectively where i and j indicate the qubits belong to the two WM operators in Fig.1 of the main text

$$M_1^i(p_i) = \begin{pmatrix} \sqrt{p_i} & 0 \\ 0 & \sqrt{1-p_i} \end{pmatrix}, \qquad M_2^j(p_j) = \begin{pmatrix} \sqrt{1-p_j} & 0 \\ 0 & \sqrt{p_j} \end{pmatrix}. \tag{1}$$

Here and after, we define $i \in \mathbb{A}, j \in \mathbb{B}, \mathbb{A} \cap \mathbb{B} = \varnothing, \mathbb{A} \cup \mathbb{B} = \{1, \ldots, N\}$, where $\mathbb{A}$ and $\mathbb{B}$ indicate a concrete combination which is composed of those qubits i and j measured by M_1^i and M_2^j separately, and $\mathbb{A}$ has k elements and $\mathbb{B}$ has $N-k$ elements, and k can be 0, 1, ..., N. p_h $(h = 1, \ldots, N)$ are WM strengths of N qubits. M_1^i and M_2^j are represented respectively as WM operators and WM is composed as $\Xi_1^i = M_1^{i\dagger} M_1^i$ and $\Xi_2^j = M_2^{j\dagger} M_2^j$, the one case of the WM operator may be $M_{l_k}^{wm} = [(M_1^i)^{\otimes k} \otimes (M_2^j)^{\otimes N-k}]_{l_k}$. Here $[\ ]_{l_k}$ indicates a concrete combination $\mathbb{A}$(or $\mathbb{B}$) of the N direct-product elements $(M_1^i)^{\otimes k} \otimes (M_2^j)^{\otimes N-k}$ in the square brackets. And the combinations of a fixed k, which come from arbitrarily changing qubits while fixing number of measurement operators M_1^i is k and fixing number of M_2^j is $N-k$, has $\mathbb{C}_N^k$ kinds, i.e., $\mathbb{A}$ (or $\mathbb{B}$) has $\mathbb{C}_N^k$ kinds combinations labeled as symbol l_k for a fixed k and $l_k = 1 \ldots \mathbb{C}_N^k$. So on the basis of this definition, we give the operational rule of $[(\ )^{\otimes k} \otimes (\ )^{\otimes N-k}]_{l_k}$ throughout our paper: combination on the direct-product elements inside the square bracket $[\ ]_{l_k}$ is the first step and calculating the direct product of the direct-product elements is the second step. If we sum over the combinations with all k, which is $\mathbb{C}_N^0 + \mathbb{C}_N^1 + \ldots + \mathbb{C}_N^N = 2^N$, so the total combinations can be 2^N cases. This WMPPF scheme includes WM before the noise channel . Here the WM is a complete measurement, i.e., $I_{2^N} = \sum_{k=0}^{N} \sum_{l_k=1}^{\mathbb{C}_N^k} M_{l_k}^{wm\dagger} M_{l_k}^{wm}$, where I_{2^N} is a identity matrix with the size 2^N . By using our scheme for each of the N qubits, according to the WM results for each qubit, we can classify our protecting scheme into 2^N cases. And each case comes from each WM result which corresponds to a combination of $(M_1^i)^{\otimes k} \otimes (M_2^j)^{\otimes N-k}$ of N qubits. Without loss of generality, here we assume the measurement result which is k qubits under M_1 measurement and $N-k$ qubits under M_1 measurement $(k = 0, 1 \ldots N)$. So that we have

$$M_{l_k}^{wm}(p_1, \ldots, p_N) = [\begin{pmatrix} \sqrt{p_i} & 0 \\ 0 & \sqrt{1-p_i} \end{pmatrix}^{\otimes k} \otimes \begin{pmatrix} \sqrt{1-p_j} & 0 \\ 0 & \sqrt{p_j} \end{pmatrix}^{\otimes N-k}]_{l_k} \tag{2a}$$

$$= [(\sqrt{p_i}|0\rangle_i\langle 0| + \sqrt{1-p_i}|1\rangle_i\langle 1|)^{\otimes k} \otimes (\sqrt{1-p_j}|0\rangle_j\langle 0| + \sqrt{p_j}|1\rangle_j\langle 1|)^{\otimes N-k}]_{l_k}. \tag{2b}$$

Here and after, the style like Eq.(2b) is a simple representation of the Eq.(2a).

In the following we operate the qubits with F_1^i and F_2^j as shown in Fig.1 of the main text. $F_1^i = I^i$ and $F_2^j = \sigma_x^j$ represent identity unitary operation and flipping operation according to the i and j of the result of the WM of M_1^i and M_2^j, respectively. Then, the N qubits are pass through the ADC which is depicted in the main text. After the ADC, F_1^i and F_2^j are used again on each qubit i and j, the same as those used before they pass into the ADC.

The WM $M_{l_k}^{wm}$ and pre-flip coupled operation is

$$\begin{aligned} M_{meas-flip} &= [\begin{pmatrix} 1 & 0 \\ 0 & 1 \end{pmatrix}^{\otimes k} \otimes \begin{pmatrix} 0 & 1 \\ 1 & 0 \end{pmatrix}^{\otimes N-k} \begin{pmatrix} \sqrt{p_i} & 0 \\ 0 & \sqrt{1-p_i} \end{pmatrix}^{\otimes k} \otimes \begin{pmatrix} \sqrt{1-p_j} & 0 \\ 0 & \sqrt{p_j} \end{pmatrix}^{\otimes N-k}]_{l_k} \\ &= [\begin{pmatrix} \sqrt{p_i} & 0 \\ 0 & \sqrt{1-p_i} \end{pmatrix}^{\otimes k} \otimes \begin{pmatrix} 0 & \sqrt{p_j} \\ \sqrt{1-p_j} & 0 \end{pmatrix}^{\otimes N-k}]_{l_k}. \end{aligned} \tag{3}$$

So, after the WM and pre-flips operation, the input generalized GHZ state $|\Phi^{in}\rangle = \alpha|0\rangle^{\otimes N} + \beta|1\rangle^{\otimes N} = \underbrace{(\alpha \cdots \beta)}_{N\ qubits}^T$ mentioned in the main text will become

$$\begin{pmatrix} \ddots & & & & & & \iddots \\ & \tilde{X} & & & & \tilde{D} & \\ & & \ddots & & \iddots & & \\ & & \iddots & & \ddots & & \\ & \tilde{C} & & & & \tilde{Y} & \\ \iddots & & & & & & \ddots \end{pmatrix}, \tag{4}$$

in which all the elements are zero except the four elements, which are $\tilde{X} = |\alpha|^2[\prod_i p_i\ |0\rangle_i\langle 0| \otimes \prod_j (1-p_j)\ |1\rangle_j\langle 1|]_{l_k}$, $\tilde{Y} = |\beta|^2[\prod_i (1-p_i)\ |1\rangle_i\langle 1| \otimes \prod_j p_j\ |0\rangle_j\langle 0|]_{l_k}$, $\tilde{C} = \alpha^*\beta\prod_h \sqrt{p_h(1-p_h)}\ [|0\rangle_i\langle 1|^{\otimes k} \otimes |1\rangle_j\langle 0|^{\otimes N-k}]_{l_k}$ and $\tilde{D} = \alpha\beta^*\prod_h \sqrt{p_h(1-p_h)}$ $[|1\rangle_i\langle 0|^{\otimes k} \otimes |0\rangle_j\langle 1|^{\otimes N-k}]_{l_k}$. In some combinations $\tilde{X}$ and $\tilde{Y}$ may locate in the down-right and up-left of the main diagonal respectively, and in these cases $\tilde{C}$ and $\tilde{D}$ will change their position to the up-right and down-left of the back-diagonal respectively. And these combinations will not affect the latter calculations. No matter what the combination l_k is, $\tilde{X}$ and $\tilde{Y}$ are always in the main diagonal, and $\tilde{C}$ and $\tilde{D}$ are always in the back-diagonal. And $\tilde{X}$, $\tilde{Y}$, $\tilde{C}$ and $\tilde{D}$ are always central symmetry in the matrix(4).

By using Kraus operators of the ADC in the main text, we can find that $|0\rangle\langle 0|$, $|1\rangle\langle 1|$, $|1\rangle\langle 0|$ and $|0\rangle\langle 1|$ evolve into $\left(\begin{smallmatrix} 1 & 0 \\ 0 & 0 \end{smallmatrix}\right)$, $\left(\begin{smallmatrix} r & 0 \\ 0 & s \end{smallmatrix}\right)$, $\left(\begin{smallmatrix} 0 & 0 \\ \sqrt{s} & 0 \end{smallmatrix}\right)$ and $\left(\begin{smallmatrix} 0 & \sqrt{s} \\ 0 & 0 \end{smallmatrix}\right)$ respectively when any qubit come out of the ADC. Note that $|1\rangle$ is high level while $|0\rangle$ is low lever, and the magnitude of the decoherence $r_h \equiv 1 - s_h$ $(h = 1, \ldots, N)$ throughout our paper. So when N-qubit come out of the ADC, $\tilde{X}$, $\tilde{Y}$, $\tilde{C}$ and $\tilde{D}$ can evolve into $\tilde{X}' = |\alpha|^2\ [\left(\begin{smallmatrix} p_i & 0 \\ 0 & 0 \end{smallmatrix}\right)^{\otimes k} \otimes \left(\begin{smallmatrix} (1-p_j)r_j & 0 \\ 0 & (1-p_j)s_j \end{smallmatrix}\right)^{\otimes N-k}]_{l_k}$, $\tilde{Y}' = |\beta|^2\ [\left(\begin{smallmatrix} (1-p_i)r_i & 0 \\ 0 & (1-p_i)s_i \end{smallmatrix}\right)^{\otimes k} \otimes \left(\begin{smallmatrix} p_j & 0 \\ 0 & 0 \end{smallmatrix}\right)^{\otimes N-k}]_{l_k}$, $\tilde{C}' = \alpha^*\beta\prod_h \sqrt{p_h(1-p_h)}\ [\left(\begin{smallmatrix} 0 & 0 \\ \sqrt{s_i} & 0 \end{smallmatrix}\right)^{\otimes k} \otimes \left(\begin{smallmatrix} 0 & \sqrt{s_j} \\ 0 & 0 \end{smallmatrix}\right)^{\otimes N-k}]_{l_k}$, and $\tilde{D}' = \alpha\beta^* \prod_h \sqrt{p_h(1-p_h)}\ [\left(\begin{smallmatrix} 0 & \sqrt{s_i} \\ 0 & 0 \end{smallmatrix}\right)^{\otimes k} \otimes \left(\begin{smallmatrix} 0 & 0 \\ \sqrt{s_j} & 0 \end{smallmatrix}\right)^{\otimes N-k}]_{l_k}$, respectively. After the post-flips , $\tilde{X}'$, $\tilde{Y}'$, $\tilde{C}'$ and $\tilde{D}'$ will become

$$\begin{aligned} X &= [[\begin{pmatrix} 1_i & 0 \\ 0 & 1_i \end{pmatrix}^{\otimes k} \otimes \begin{pmatrix} 0 & 1_j \\ 1_j & 0 \end{pmatrix}^{\otimes N-k}]\ \tilde{X}'\ [\begin{pmatrix} 1_i & 0 \\ 0 & 1_i \end{pmatrix}^{\otimes k} \otimes \begin{pmatrix} 0 & 1_j \\ 1_j & 0 \end{pmatrix}^{\otimes N-k}]]_{l_k} \\ &= [[\begin{pmatrix} 1_i & 0 \\ 0 & 1_i \end{pmatrix}^{\otimes k} \otimes \begin{pmatrix} 0 & 1_j \\ 1_j & 0 \end{pmatrix}^{\otimes N-k}][|\alpha|^2 \begin{pmatrix} p_i & 0 \\ 0 & 0 \end{pmatrix}^{\otimes k} \otimes \begin{pmatrix} (1-p_j)r_j & 0 \\ 0 & (1-p_j)s_j \end{pmatrix}^{\otimes N-k}] \\ &\quad [\begin{pmatrix} 1_i & 0 \\ 0 & 1_i \end{pmatrix}^{\otimes k} \otimes \begin{pmatrix} 0 & 1_j \\ 1_j & 0 \end{pmatrix}^{\otimes N-k}]]_{l_k} \\ &= |\alpha|^2[\begin{pmatrix} p_i & 0 \\ 0 & 0 \end{pmatrix}^{\otimes k} \otimes \begin{pmatrix} (1-p_j)s_j & 0 \\ 0 & (1-p_j)r_j \end{pmatrix}^{\otimes N-k}]_{l_k}, \end{aligned} \tag{5}$$

$$
\begin{aligned}
Y = &[[\begin{pmatrix} 1_i & 0 \\ 0 & 1_i \end{pmatrix}^{\otimes k} \otimes \begin{pmatrix} 0 & 1_j \\ 1_j & 0 \end{pmatrix}^{\otimes N-k}][|\beta|^2 \begin{pmatrix} (1-p_i)r_i & 0 \\ 0 & (1-p_i)s_i \end{pmatrix}^{\otimes k} \otimes \begin{pmatrix} p_j & 0 \\ 0 & 0 \end{pmatrix}^{\otimes N-k}] \\
&[\begin{pmatrix} 1_i & 0 \\ 0 & 1_i \end{pmatrix}^{\otimes k} \otimes \begin{pmatrix} 0 & 1_j \\ 1_j & 0 \end{pmatrix}^{\otimes N-k}]]_{l_k} \\
&= |\beta|^2 [\begin{pmatrix} (1-p_i)r_i & 0 \\ 0 & (1-p_i)s_i \end{pmatrix}^{\otimes k} \otimes \begin{pmatrix} 0 & 0 \\ 0 & p_j \end{pmatrix}^{\otimes N-k}]_{l_k},
\end{aligned} \tag{6}
$$

$$
\begin{aligned}
C = &\alpha^* \beta \prod_h \sqrt{p_h(1-p_h)}\, [[\begin{pmatrix} 1_i & 0 \\ 0 & 1_i \end{pmatrix}^{\otimes k} \otimes \begin{pmatrix} 0 & 1_j \\ 1_j & 0 \end{pmatrix}^{\otimes N-k}] \\
&[\begin{pmatrix} 0 & \\ \sqrt{s_i} & 0 \end{pmatrix}^{\otimes k} \otimes \begin{pmatrix} 0 & \sqrt{s_j} \\ 0 & 0 \end{pmatrix}^{\otimes N-k}][\begin{pmatrix} 1_i & 0 \\ 0 & 1_i \end{pmatrix}^{\otimes k} \otimes \begin{pmatrix} 0 & 1_j \\ 1_j & 0 \end{pmatrix}^{\otimes N-k}]]_{l_k} \\
&= \alpha^* \beta \begin{pmatrix} 0 & 0 \\ \sqrt{p_h(1-p_h)s_h} & 0 \end{pmatrix}^{\otimes N},
\end{aligned} \tag{7}
$$

and

$$
\begin{aligned}
D = &\alpha \beta^* \prod_h \sqrt{p_h(1-p_h)}\, [[\begin{pmatrix} 1_i & 0 \\ 0 & 1_i \end{pmatrix}^{\otimes k} \otimes \begin{pmatrix} 0 & 1_j \\ 1_j & 0 \end{pmatrix}^{\otimes N-k}] \\
&[\begin{pmatrix} 0 & \sqrt{s_i} \\ 0 & 0 \end{pmatrix}^{\otimes k} \otimes \begin{pmatrix} 0 & 0 \\ \sqrt{s_j} & 0 \end{pmatrix}^{\otimes N-k}][\begin{pmatrix} 1_i & 0 \\ 0 & 1_i \end{pmatrix}^{\otimes k} \otimes \begin{pmatrix} 0 & 1_j \\ 1_j & 0 \end{pmatrix}^{\otimes N-k}]]_{l_k} \\
&= \alpha \beta^* \begin{pmatrix} 0 & \sqrt{p_h(1-p_h)s_h} \\ 0 & 0 \end{pmatrix}^{\otimes N},
\end{aligned} \tag{8}
$$

respectively.

So after passing the noise channel, in each case, the final density matrix can be derived as $\tilde{\rho}$, where $\tilde{\rho}= \varepsilon(\rho^{in})$ for N-qubits. Here $\rho^{in}= \rho^{GGHZ}$ and the dissipation channel ε is ADC. we can normalize the $\tilde{\rho}$ into ρ^{out} and $\rho^{out} = \frac{1}{P}\tilde{\rho}$, which means that the generalized GHZ state ρ^{GGHZ} evolves into ρ^{out} with the probability P where

$$
\rho^{out} = \frac{1}{P}\tilde{\rho} = \frac{1}{P}\begin{pmatrix} A & \mathbf{0} & D \\ \mathbf{0} & E & \mathbf{0} \\ C & \mathbf{0} & B \end{pmatrix} = \rho_1 \oplus E, \tag{9}
$$

$P = A + B + Tr(E)$ and

$$
\rho_1 = \frac{1}{P}\begin{pmatrix} A & D \\ C & B \end{pmatrix}. \tag{10}
$$

Here the bases of $A, B, C,$ and D are $|0\rangle\langle 0|^{\otimes N}$, $|1\rangle\langle 1|^{\otimes N}$, $|1\rangle\langle 0|^{\otimes N}$ and $|0\rangle\langle 1|^{\otimes N}$ respectively which are values of the four corners of the $\tilde{\rho}$. And $D \equiv C^*$. E is a diagonal matrix with its elements' bases are $|K(bin)\rangle\langle K(bin)|$, where $K = 1, 2, \dots, 2^N - 2$ and $K(bin)$ is the binary representation of K with N qubits. Here k indicates the result of the WM and means k qubits are measured by E_1 and $N-k$ qubits are measured by M_2. Here A, B and E are independent with ϕ. C and D are invariant due to different measurement results and they are the only two part which is dependent of ϕ according to the whole ρ^{out}. So if we can diagonalize ρ_1, then we can calculate the average QFI, average fidelity and probability.

After the phase gates, the initial generalized GHZ state can get an extra $e^{iN\phi}$ in the basis $|1\rangle^{\otimes N}$, and note that ϕ can be any values. In this paper, we calculate the average QFIs, average fidelities and probabilities with popular formulas which are effective for the situation that all qubits may (or may not) have different parameters (e.g., for two qubits situation, when the two channels' magnitude of the decoherence $r_1 \neq r_2$, it is necessary to use the popular formulas). And after that we use $\overset{S}{=}$ to indicate the situation that all qubits have same pre- and post-measurement parameters and same decaying parameters, which can make our calculations simplified.

When $k = 0$ (i.e., all qubits flip), there is only one combination ($\mathbb{C}_N^0 = 1$) because of only one case. Here and after we use $\mathbb{C}_N^k = \frac{N!}{k!(N-k)!}$ to indicate combination number for the situation where k qubits are measured by M_1 and $N-k$ qubits

are measured by M_2, $A_0=[\prod_h(1-p_h)\ s_h]\ |\alpha|^2|0\rangle\langle 0|^{\otimes N} \stackrel{S}{=} (1-p)^N\ s^N\ |\alpha|^2|0\rangle\langle 0|^{\otimes N}$, $B_0=[\prod_h(1-p_h)\ |\alpha|^2\ r_h + \prod_h p_h|\beta|^2\ ]\ |1\rangle\langle 1|^{\otimes N} \stackrel{S}{=} [(1-p)^N\ |\alpha|^2\ r^N + p^N|\beta|^2\ ]\ |1\rangle\langle 1|^{\otimes N}$. $C_0=D_0^\dagger=\alpha^*\beta\ \prod_h \sqrt{p_h}\ \sqrt{1-p_h}\ \sqrt{s_h}\ |1\rangle\langle 0|^{\otimes N} \stackrel{S}{=} \alpha^*\beta\ \sqrt{p}^N\ \sqrt{1-p}^N\ \sqrt{s}^N\ |1\rangle\langle 0|^{\otimes N}$. $E_0=|\alpha|^2\ [\ [(1-p_h)s_h|0\rangle_h\langle 0| + (1-p_h)r_h|1\rangle_h\langle 1|]^{\otimes N} - [\prod_h(1-p_h)s_h]\ |0\rangle\langle 0|^{\otimes N} - [\prod_h(1-p_h)\ r_h]\ |1\rangle\langle 1|^{\otimes N}] \stackrel{S}{=} (1-p)^N\ |\alpha|^2\ [(s|0\rangle_h\langle 0| + r|1\rangle_h\langle 1|)^{\otimes N} - s^N\ |0\rangle\langle 0|^{\otimes N} - r^N\ |1\rangle\langle 1|^{\otimes N}]$. $P_0=Tr(\ A_0+B_0+E_0)=|\alpha|^2\ \prod_h(1-p_h) + |\beta|^2\ \prod_h p_h \stackrel{S}{=} |\alpha|^2\ (1-p)^N + |\beta|^2 p^N$.

Here and after, to simplify the expression and avoid double counting by the product operation of $\prod_h$, we omit h in $|0\rangle_h\langle 0|^{\otimes N}$, $|1\rangle_h\langle 1|^{\otimes N}$, $|0\rangle_h\langle 1|^{\otimes N}$ and $|1\rangle_h\langle 0|^{\otimes N}$ as $|0\rangle\langle 0|^{\otimes N}$, $|1\rangle\langle 1|^{\otimes N}$, $|0\rangle\langle 1|^{\otimes N}$ and $|1\rangle\langle 0|^{\otimes N}$, respectively.

When $k=1$ to $N-1$ (i.e., $N-k$ qubits flip), there are $\mathbb{C}_N^k$ kind cases (or combinations, we use l_k as superscript to indicate them below and $l_k=1\ldots\mathbb{C}_N^k$) for a certain value k. So for $k=1$ to $N-1$, the total number of the cases is $\mathbb{C}_N^1+\mathbb{C}_N^2+\ldots+\mathbb{C}_N^{N-1}=2^N-2$. Let us discuss the matrix of ρ under this situation, in which $A_{l_k}=|\alpha|^2\ [\prod_i p_i \prod_j (1-p_j)\ s_j]_{l_k}\ |0\rangle\langle 0|^{\otimes N} \stackrel{S}{=} |\alpha|^2\ p^k\ (1-p)^{N-k}\ s^{N-k}\ |0\rangle\langle 0|^N$, $B_{l_k}=|\beta|^2\ [\prod_i (1-p_i)\ s_i \prod_j p_j\ ]_{l_k}\ |1\rangle\langle 1|^{\otimes N} \stackrel{S}{=} |\beta|^2\ (1-p)^k\ s^k\ p^{N-k}\ |1\rangle\langle 1|^{\ \otimes N}$ and $C_{l_k}=D_{l_k}^\dagger=[\ \alpha^*\beta\ \prod_h\sqrt{p_h(1-p_h)s_h}\ ]\ |1\rangle\langle 0|^{\otimes N} \stackrel{S}{=} \alpha^*\beta\ \sqrt{p(1-p)s}^N\ |1\rangle\langle 0|^{\otimes N}$ are dependent with k but independent of l_k. However, $E_{l_k}=|\alpha|^2\ [\ [p_i\ |0\rangle_i\langle 0|]^{\otimes k} \otimes [(1-p_j)\ s_j|0\rangle_j\langle 0| + (1-p_j)\ r_j|1\rangle_j\langle 1|]^{\otimes N-k}]_{l_k} + |\beta|^2\ [\ [(1-p_i)r_i\ |0\rangle_i\langle 0| + (1-p_i)s_i|1\rangle_i\langle 1|]^{\otimes k} \otimes [p_j\ |1\rangle_j\langle 1|]^{\otimes N-k}]_{l_k} - A_{l_k} - B_{l_k} \stackrel{S}{=} p^k\ (1-p)^{N-k}\ |\alpha|^2\ [\ |0\rangle_i\langle 0|^{\otimes k} \otimes [s|0\rangle_j\langle 0| + r|1\rangle_j\langle 1|]^{\otimes N-k}]_{l_k} + (1-p)^k\ p^{N-k}\ |\beta|^2 \otimes [(r\ |0\rangle_i\langle 0| + s|1\rangle_i\langle 1|)^{\otimes k} \otimes |1\rangle_j\langle 1|^{\otimes N-k}]_{l_k} - A_{l_k} - B_{l_k}$. Note that as the former here the $[\ \ ]_{l_k}$ means the combination l_k for the N qubits in $[\ \ ]$, $l_k=1\ \ldots\ \mathbb{C}_N^k$, no matter what states the qubits $\{i\}$ and $\{j\}$ are, e.g., $|0\rangle_i\langle 0|$ or $(s_j|0\rangle_j\langle 0| + r_j|1\rangle_j\langle 1|)$ or $(r_j\ |0\rangle_j\langle 0| + s|1\rangle_j\langle 1|)$. And as mentioned before, the combination operation is before the direct product still. $P_{l_k}=Tr(\ A_{l_k}+B_{l_k}+E_{l_k})=[|\alpha|^2\ \prod_i p_i \prod_j(1-p_j) + |\beta|^2\ \prod_i (1-p_i)\ \prod_j p_j]_{l_k} \stackrel{S}{=} p^k(1-p)^{N-k}\ |\alpha|^2 + (1-p)^k\ p^{N-k}\ |\beta|^2$.

When $k=N$ (i.e., no qubit flips), $A_N=[|\alpha|^2\ \prod_h p_h + |\beta|^2\ \prod_h (1-p_h)\ r_h]\ |0\rangle\langle 0|^{\otimes N} \stackrel{S}{=} [|\alpha|^2 p^N + |\beta|^2(1-p)^N\ r^N]\ |0\rangle\langle 0|^{\otimes N}$, $B_N=|\beta|^2\ [\prod_h (1-p_h)\ s_h]|1\rangle\langle 1|^{\otimes N} \stackrel{S}{=} |\beta|^2\ (1-p)^N\ s^N\ |1\rangle\langle 1|^{\otimes N}$, $C_N=D_N^\dagger=\alpha^*\beta\ \prod_h\sqrt{p_h}\ \sqrt{1-p_h}\ \sqrt{s_h}\ |1\rangle\langle 0|^{\otimes N} \stackrel{S}{=} \alpha^*\beta\ \sqrt{p}^N\ \sqrt{1-p}^N\ \sqrt{s}^N\ |1\rangle\langle 0|^{\otimes N}$. $E_N=|\beta|^2\ [\ ((1-p_h)r_h\ |0\rangle_h\langle 0| + (1-p_h)s_h|1\rangle_h\langle 1|)^{\ \otimes N} - \prod_h\ (1-p_h)r_h\ |0\rangle\langle 0|^{\otimes N} - \prod_h\ (1-p_h)\ s_h\ |1\rangle\langle 1|^{\otimes N}] \stackrel{S}{=} |\beta|^2\ (1-p)^N\ [(r\ |0\rangle_h\langle 0| + s|1\rangle_h\langle 1|)^{\otimes N} - r^N\ |0\rangle\langle 0|^{\otimes N} - s^N\ |1\rangle\langle 1|^{\otimes N}]$. $P_N=Tr(\ A_N+B_N+E_N)=|\alpha|^2\ [\prod_h p_h\ ] + |\beta|^2\ [\prod_h (1-p_h)] \stackrel{S}{=} |\alpha|^2 p^N + |\beta|^2\ (1-p)^N$, which indicates the trace of $\tilde{\rho}$.

By using Eq.(16) in the **Methods** of the main text, the QFI can be calculated as $F_0=\frac{1}{P_0}\ \frac{4|C_0|^2N^2}{A_0+B_0}$, $F_{l_k}=\frac{1}{P_{l_k}}\ \frac{4\ |C_k|^2N^2}{A_{l_k}+B_{l_k}}$, $F_N=\frac{1}{P_N}\ \frac{4|C_N|^2N^2}{A_N+B_N}$, with probability P_0, P_{l_k}, and P_N, seperately. Note that here A_0, B_0 and C_0 are the corresponding A, B, C in Eq.(9) which is the case that all qubits flip; A_{l_k}, B_{l_k} and C_{l_k} are the corresponding A, B, C in Eq.(9) which is the case that $N-k$ qubits flip; A_N, B_N and C_N are the corresponding A, B, C in Eq.(9) which is the case that no qubits flip. These nine values just mentioned above do not contain their bases, and for calculation, we only need consider their coefficient just as Eq.(16) of the

Methods shows. So, the total average QFI can be analytically gotten with

$$\begin{aligned}
F_{WMPPF} &= P_0F_0 + P_NF_N + \sum_{k=1}^{N-1}\sum_{l_k=1}^{\mathbb{C}_N^k} P_{l_k}F_{l_k} \\
&= \frac{4|C_0|^2N^2}{A_0+B_0} + \frac{4|C_N|^2N^2}{A_N+B_N} + \sum_{k=1}^{N-1}\sum_{l_k=1}^{\mathbb{C}_N^k} \frac{4\,|C_{l_k}|^2N^2}{A_{l_k}+B_{l_k}} \\
&= \frac{4|\alpha\beta|^2N^2\prod_h p_h(1-p_h)s_h}{|\alpha|^2\prod_h(1-p_h)s_h + [|\alpha|^2\prod_h(1-p_h)r_h + |\beta|^2\prod_h p_h]} + \frac{4|\alpha\beta|^2N^2\prod_h p_h(1-p_h)s_h}{[|\alpha|^2\prod_h p_h + |\beta|^2\prod_h(1-p_h)r_h] + |\beta|^2\prod_h(1-p_h)s_h} \\
&\quad + \sum_{k=1}^{N-1}\sum_{l_k=1}^{\mathbb{C}_N^k} \frac{4\,|\alpha\beta|^2N^2\prod_h p_h(1-p_h)s_h}{|\alpha|^2[\prod_i p_i\prod_j(1-p_j)s_j]_{l_k} + |\beta|^2[\prod_i(1-p_i)s_i\prod_j p_j]_{l_k}} \\
&\overset{S}{=} \frac{4|C_0|^2N^2}{A_0+B_0} + \frac{4|C_N|^2N^2}{A_N+B_N} + \sum_{k=1}^{N-1}\mathbb{C}_N^k\frac{4\,|C_{l_k}|^2N^2}{A_{l_k}+B_{l_k}}
\end{aligned} \tag{11a}$$

$$\begin{aligned}
&\overset{S}{=} \frac{4|\alpha\beta|^2N^2p^N(1-p)^Ns^N}{|\alpha|^2((1-p)s)^N + [|\alpha|^2((1-p)r)^N + |\beta|^2p^N]} + \frac{4|\alpha\beta|^2N^2p^N(1-p)^Ns^N}{[|\alpha|^2p^N + |\beta|^2((1-p)r)^N] + |\beta|^2((1-p)s)^N} \\
&\quad + \sum_{k=1}^{N-1}\mathbb{C}_N^k\frac{4\,|\alpha\beta|^2N^2p^N(1-p)^Ns^N}{|\alpha|^2[p^k((1-p)s)^{N-k}] + |\beta|^2[((1-p)s)^kp^{N-k}]}.
\end{aligned} \tag{11b}$$

Here note that if we choose all the qubits with same weak measurement parameters and same ADC parameters, i.e. under $\overset{S}{=}$ situation, A_{l_k}, B_{l_k}, C_{l_k} and P_{l_k} which we use here and latter have the same values for different combinations l_k, respectively. And the value of them just depend on k but do not depend on l_k In other words, according to A_{l_k}, B_{l_k}, C_{l_k} and P_{l_k}, each of which has l_k combinations ($l_k = 1\ldots\mathbb{C}_N^k$) with the same k, have the same function values for $\mathbb{C}_N^k$ kinds of combinations respectively. And thus in our above summation, e.g., $\overset{S}{=}\sum_{k=1}^{N-1}\mathbb{C}_N^k\frac{4\,|C_{l_k}|^2N^2}{A_{l_k}+B_{l_k}}$, for simplicity, we still keep the tag l_k in the right part of the $\overset{S}{=}$, but all the parameters A_{l_k}, B_{l_k}, C_{l_k} should be used with simplified ones. So throughout our paper, when in an expression $\overset{S}{=}$ emerges, we should substitute the simplified parameters into it for the further calculation. Besides, as we have seen, the value of the simplified expression is dependent with k but independent of l_k even if its parameters are tagged by l_k. And it is the same way to handle the other similar summations of our paper. Note that sometimes even under the $\overset{S}{=}$, the bases of the expression may still depend on the combination l_k, e.g., E_{l_k} in the above.

The total probability of WMPPF is

$$\begin{aligned}
P_{WMPPF} &= P_0 + P_N + \sum_{k=1}^{N-1}\sum_{l_k=1}^{\mathbb{C}_N^k} P_{l_k} \\
&= |\alpha|^2\prod_h[p_h + (1-p_h)(s_h+r_h)] + |\beta|^2\prod_h[(1-p_h)(s_h+r_h) + p_h] \\
&\equiv 1.
\end{aligned} \tag{12}$$

The average fidelity of WMPPF can be give by

$$Fid = \langle\Phi|\rho|\Phi\rangle = \frac{1}{P}(|\alpha|^2A + \alpha\beta^*C + \alpha^*\beta D + |\beta|^2B). \tag{13}$$

Similar as above discussion, the different cases can get different average fidelities which are Fid_0, Fid_N and Fid_{l_k}, where the probability is as the same with mentioned above.

$$\begin{aligned}
Fid_0 &= \frac{1}{P_0}(|\alpha|^2A_0 + \alpha\beta^*C_0 + \alpha^*\beta D_0 + |\beta|^2B_0); \\
Fid_N &= \frac{1}{P_N}(|\alpha|^2A_N + \alpha\beta^*C_N + \alpha^*\beta D_N + |\beta|^2B_N); \\
Fid_{l_k} &= \frac{1}{P_{l_k}}(|\alpha|^2A_{l_k} + \alpha\beta^*C_{l_k} + \alpha^*\beta D_{l_k} + |\beta|^2B_{l_k}).
\end{aligned} \tag{14}$$

Thus the average fidelity of WMPPF is

$$
\begin{aligned}
&Fid_{WMPPF}\\
&=\frac{1}{P_{WMPPF}}[P_0Fid_0+P_NFid_N+\textstyle\sum_{k=1}^{N-1}\sum_{l_k=1}^{\mathbb{C}_N^k}P_{l_k}Fid_{l_k}]\\
&=\frac{1}{P_{WMPPF}}[(|\alpha|^2A_0+\alpha\beta^*C_0+\alpha^*\beta D_0+|\beta|^2B_0)+(|\alpha|^2A_N+\alpha\beta^*C_N+\alpha^*\beta D_N+|\beta|^2B_N)\\
&\quad+\textstyle\sum_{k=1}^{N-1}\sum_{l_k=1}^{\mathbb{C}_N^k}(|\alpha|^2A_{l_k}+\alpha\beta^*C_{l_k}+\alpha^*\beta D_{l_k}+|\beta|^2B_{l_k})]\\
&=\frac{1}{P_{WMPPF}}[|\alpha|^4\prod_h[p_h+(1-p_h)s_h]+2|\alpha\beta|^2\prod_h(1-p_h)r_h+|\beta|^4\prod_h[p_h+(1-p_h)s_h]\\
&\quad+2^{N+1}|\alpha\beta|^2\prod_h(s_hp_h(1-p_h))^{1/2}]\\
&\overset{S}{=}\frac{1}{P_{WMPPF}}[(|\alpha|^2A_0+\alpha\beta^*C_0+\alpha^*\beta D_0+|\beta|^2B_0)+(|\alpha|^2A_N+\alpha\beta^*C_N+\alpha^*\beta D_N+|\beta|^2B_N)\\
&\quad+\textstyle\sum_{k=1}^{N-1}\mathbb{C}_N^k(|\alpha|^2A_{l_k}+\alpha\beta^*C_{l_k}+\alpha^*\beta D_{l_k}+|\beta|^2B_{l_k})]\\
&\overset{S}{=}\frac{1}{P_{WMPPF}}\{|\alpha|^4[p+(1-p)s]^N+2|\alpha\beta|^2(1-p)^Nr^N+|\beta|^4[p+(1-p)s]^N\\
&\qquad+2^{N+1}|\alpha\beta|^2(sp(1-p))^{N/2}\},
\end{aligned}
\tag{15}
$$

where in this equation $P_{WMPPF}\equiv 1$.

Based on the formulas discussed above, if we provide $\theta=\pi/2$ and $\phi_0=0$ which means that our initial state generalized GHZ state becomes N-qubit GHZ state. Therefore, these formulas can be used to discuss GHZ state and generalized GHZ states as we will see in the main text.

The calculations of the output ρ^{out}, average QFI of phase and average fidelity for WMQMR. In the main text, we have given the whole processing of WMQMR and the WM operator M^{wm}. So for N qubits, we have

$$
M^{wm}=\begin{pmatrix}1 & 0\\ 0 & \sqrt{1-p_{1h}}\end{pmatrix}^{\otimes N},
\tag{16}
$$

and h= $1,2,\ldots,N$ as we have defined before.

So, after the WM, the input generalized GHZ state, which is the same as that in WMPPF in the main text: $|\Phi^{in}\rangle=\alpha|0\rangle^{\otimes N}$ $+\beta|1\rangle^{\otimes N}$= $\underbrace{(\alpha\cdots\beta)^T}_{N\ qubits}$, will become

$$
\begin{pmatrix}\tilde{X} & & & \tilde{D}\\ & \ddots & \iddots & \\ & \iddots & \ddots & \\ \tilde{C} & & & \tilde{Y}\end{pmatrix},
\tag{17}
$$

in which only four elements are not zeroes while others are zeroes, and the four elements are $\tilde{X}=|\alpha|^2\ |0\rangle\langle 0|^{\otimes N}$, $\tilde{Y}=|\beta|^2[\prod_h(1-p_{1h})]\ |1\rangle\langle 1|^{\otimes N}$, $\tilde{C}=\alpha^*\beta[\prod_h\sqrt{1-p_{1h}}\,]\ |1\rangle\langle 0|^{\otimes N}$ and $\tilde{D}=\alpha\beta^*[\prod_h\sqrt{1-p_{1h}}\,]\ |0\rangle\langle 1|^{\otimes N}$. By using Kraus operators of the ADC in the main text, when the N qubits come out of the ADC, we can find that $\tilde{X}$, $\tilde{Y}$, $\tilde{C}$ and $\tilde{D}$ can evolve into four states $\tilde{X}'=|\alpha|^2\ (\begin{smallmatrix}1&0\\0&0\end{smallmatrix})^{\otimes N}$, $\tilde{Y}'$ =$|\beta|^2\ (\begin{smallmatrix}(1-p_{1h})r_h & 0\\ 0 & (1-p_{1h})s_h\end{smallmatrix})^{\otimes N}$, $\tilde{C}'$ =$\alpha^*\beta\ (\begin{smallmatrix}0 & 0\\ \sqrt{(1-p_{1h})s_h} & 0\end{smallmatrix})^{\otimes N}$, and $\tilde{D}'$ =$\alpha\beta^*\ (\begin{smallmatrix}0 & \sqrt{(1-p_{1h})s_h}\\ 0 & 0\end{smallmatrix})^{\otimes N}$, respectively. After the reversing measurement $M^{rev}=(\begin{smallmatrix}\sqrt{1-p_{rh}} & 0\\ 0 & 1\end{smallmatrix})^{\otimes N}$, $\tilde{X}'$, $\tilde{Y}'$, $\tilde{C}'$ and $\tilde{D}'$ will become

$$
\begin{aligned}
&X=M^{rev}\ \tilde{X}'\ M^{rev\dagger}\\
&=|\alpha|^2\begin{pmatrix}(1-p_{1h})(1-p_{rh}) & 0\\ 0 & 0\end{pmatrix}^{\otimes N},
\end{aligned}
\tag{18}
$$

$$
\begin{aligned}
Y &= M^{rev}\ \tilde{Y}'\ M^{rev\dagger} \\
&= |\beta|^2 \begin{pmatrix} (1-p_{1h})(1-p_{rh})r_h & 0 \\ 0 & (1-p_{1h})s_h \end{pmatrix}^{\otimes N},
\end{aligned} \tag{19}
$$

$$
\begin{aligned}
C &= M^{rev}\ \tilde{C}'\ M^{rev\dagger} \\
&= \alpha^*\beta \begin{pmatrix} 0 & 0 \\ \sqrt{s_h(1-p_{1h})(1-p_{rh})} & 0 \end{pmatrix}^{\otimes N}
\end{aligned} \tag{20}
$$

and

$$
\begin{aligned}
D &= M^{rev}\ \tilde{D}'\ M^{rev\dagger} \\
&= \alpha\beta^* \begin{pmatrix} 0 & \sqrt{s_h(1-p_{1h})(1-p_{rh})} \\ 0 & 0 \end{pmatrix}^{\otimes N}
\end{aligned} \tag{21}
$$

respectively, the same structure of evolution matrix as that derived for WMPPF.

So, at last the output matrix $\tilde{\rho}=\varepsilon(\rho^{in})=X+Y+C+D$ which is not a normalized matrix. And the dissipation channel ε is ADC. It is easy to find that $\tilde{\rho}$ is diagonal except two elements C and D which are in the two corners of $\tilde{\rho}$.

The normalized output matrix of WMQMR for N-qubits is like the style of WMPPF: $\rho^{out}=\frac{1}{P_{WMQMR}}\tilde{\rho}=\frac{1}{P_{WMQMR}}\varepsilon(\rho^{GGHZ})=\rho_1\bigoplus E$. Here we have substitute ρ^{GGHZ} into ρ^{in}. So

$$
\rho^{out}=\frac{1}{P_{WMQMR}}\tilde{\rho}=\frac{1}{P_{WMQMR}}\begin{pmatrix} A & \mathbf{0} & D \\ \mathbf{0} & E & \mathbf{0} \\ C & \mathbf{0} & B \end{pmatrix}, \tag{22}
$$

and P_{WMQMR} is the trace of $\tilde{\rho}$ which means the probability of ρ^{out}, and

$$
\rho_1=\frac{1}{P_{WMQMR}}\begin{pmatrix} A & D \\ C & B \end{pmatrix}, \tag{23}
$$

here

$$
\begin{aligned}
A &= |\alpha|^2\prod_h(1-p_{rh})+|\beta|^2\prod_h[(1-p_{1h})r_h(1-p_{rh})] \overset{S}{=} |\alpha|^2(1-p_r)^N+(1-p_1)^N|\beta|^2r^N(1-p_r)^N; \\
B &= |\beta|^2\prod_h(1-p_{1h})s_h \overset{S}{=} (1-p_1)^N|\beta|^2s^N; \\
C &= \alpha^*\beta\prod_h(1-p_{1h})^{\frac{1}{2}}s_h^{\frac{1}{2}}(1-p_{rh})^{\frac{1}{2}} \overset{S}{=} \alpha^*\beta(1-p_1)^{\frac{N}{2}}s^{\frac{N}{2}}(1-p_r)^{\frac{N}{2}}; \\
D &= \alpha\beta^*\prod_h(1-p_{1h})^{\frac{1}{2}}s_h^{\frac{1}{2}}(1-p_{rh})^{\frac{1}{2}} \overset{S}{=} \alpha\beta^*(1-p_1)^{\frac{N}{2}}s^{\frac{N}{2}}(1-p_r)^{\frac{N}{2}}; \\
E &= (1-p_1)^N|\beta|^2\sum_{m=1}^{N-1}\sum_{l_m=1}^{C_N^m}\left[\prod_{i=1}^{N-m}r_i(1-p_{ri})|0\rangle_i\langle 0|\prod_{j=1}^{m}s_j|1\rangle_j\langle 1|\right]_{l_m} \\
&\overset{S}{=} (1-p_1)^N|\beta|^2\sum_{m=1}^{N-1}r^{N-m}(1-p_r)^{N-m}s^m\sum_{l_m=1}^{C_N^m}\left[|0\rangle_i\langle 0|^{\otimes N-m}|1\rangle_j\langle 1|^{\otimes m}\right]_{l_m}; \\
P_{WMQMR} &= Tr[\tilde{\rho}]=A+B+Tr(E)=|\alpha|^2\prod_h(1-p_{rh})+|\beta|^2\prod_h(1-p_{1h})(r_h(1-p_{rh})+s_h) \\
&\overset{S}{=} |\alpha|^2(1-p_r)^N+|\beta|^2(1-p_1)^N(r(1-p_r)+s)^N.
\end{aligned} \tag{24}
$$

Here the bases of A, B, C, and D are $|0\rangle\langle 0|^{\otimes N}$, $|1\rangle\langle 1|^{\otimes N}$, $|1\rangle\langle 0|^{\otimes N}$ and $|0\rangle\langle 1|^{\otimes N}$ respectively. And here i and j construct the sets $\{i\}$ and $\{j\}$which have $N-m$ and m elements separately, and $\{i\}\cap\{j\}=\varnothing$, $\{i\}\cup\{j\}=\{1,2,3,\ldots,N\}$. l_m indicates

a concrete combination for a fixed m which indicates a concrete diagonal element of the $\tilde{\rho}$ except two ones whose bases are $|0\rangle\langle 0|^{\otimes N}$ and $|1\rangle\langle 1|^{\otimes N}$. And with the ρ^{out} we can calculate the average QFI and average fidelity:

$$F_{WMQMR}=4|C|^2N^2/(A+B) \quad = \frac{4|\alpha\beta|^2\prod_h(1-r_h)N^2}{\frac{|\alpha|^2}{\prod_h(1-p_{1h})}+|\beta|^2(\frac{\prod_h s_h}{\prod_h(1-p_{rh})}+\prod_h r_h)} \overset{S}{=} \frac{4|\alpha\beta|^2(1-r)^N N^2}{\frac{|\alpha|^2}{(1-p_1)^N}+|\beta|^2(\frac{(1-r)^N}{(1-p_r)^N}+r^N)},$$

$$\begin{aligned}Fid_{WMQMR}=&\frac{1}{P_{WMQMR}}[|\alpha|^4\prod_h(1-p_{rh})^N+|\alpha\beta|^2\prod_h(1-p_{1h})r_h(1-p_{rh})+2|\alpha\beta|^2\prod_h(1-p_{1h})^{\frac{1}{2}}s_h^{\frac{1}{2}}(1-p_{rh})^{\frac{1}{2}}\\&+|\beta|^4\prod_h(1-p_{1h})s_h]\\ \overset{S}{=}&\frac{1}{P_{WMQMR}}[|\alpha|^4(1-p_r)^N+|\alpha\beta|^2(1-p_1)^N r^N(1-p_r)^N+2|\alpha\beta|^2(1-p_1)^{\frac{N}{2}}s^{\frac{N}{2}}(1-p_r)^{\frac{N}{2}}\\&+|\beta|^4(1-p_1)^N s^N].\end{aligned}$$

Average QFIs of weight factor for WMQMR and DN. In this section, we discuss average QFI of weight factor for WMQMR and QFI of weight factor for DN, where both initial states are generalized GHZ state $|\Phi^{in}\rangle$ mentioned in the former. Following the discussion of the previous section on calculating the ρ^{out} which is the evolved normalized matrix for WMQMR, based on this ρ^{out}, below we will continue to discuss the QFI of the weight factor (i.e., amplitude parameter) θ to the WMQMR case. By using the general QFI formula according to θ, we have the average QFI of θ for WMQMR, which is

$$F^{\theta}_{WMQMR}=P_{WMQMR}[\sum_{l'}\frac{(\partial_\theta\lambda_{l'})^2}{\lambda_{l'}}+\sum_{l\neq m}\frac{2(\lambda_l-\lambda_m)^2}{\lambda_l+\lambda_m}|\langle\varphi_l|\partial_\theta|\varphi_m\rangle|^2] \tag{25}$$

$$\begin{aligned}=&P_{WMQMR}[\frac{(\frac{\partial\lambda_1}{\partial\theta})^2}{\lambda_1}+\frac{(\frac{\partial\lambda_2}{\partial\theta})^2}{\lambda_2}]+P^2_{WMQMR}\sum_{m=1}^{N-1}\frac{(\frac{\partial(|\beta|^2/P_{WMQMR})}{\partial\theta})^2}{|\beta|^2}\prod_{h=1}^{N}(1-p_{1h})\sum_{l_m=1}^{C_N^m}\left[\prod_{i=1}^{N-m}r_i(1-p_{ri})\prod_{j=1}^{m}s_j\right]_{l_m}\\&+P^2_{WMQMR}\frac{4\left[(\partial(\frac{|C|}{P_{WMQMR}})/\partial\theta)(A-B)-|C|(\partial(\frac{A-B}{P_{WMQMR}})/\partial\theta)\right]^2}{(A+B)[(A-B)^2+4|C|^2]}\end{aligned} \tag{26}$$

$$\begin{aligned}\overset{S}{=}&P_{WMQMR}[\frac{(\frac{\partial\lambda_1}{\partial\theta})^2}{\lambda_1}+\frac{(\frac{\partial\lambda_2}{\partial\theta})^2}{\lambda_2}]+P^2_{WMQMR}\sum_{m=1}^{N-1}C_N^m\frac{(\frac{\partial(|\beta|^2/P_{WMQMR})}{\partial\theta})^2}{|\beta|^2}(1-p_1)^N(1-p_r)^{N-m}r^{N-m}s^m\\&+P^2_{WMQMR}\frac{4\left[(\partial(\frac{|C|}{P_{WMQMR}})/\partial\theta)(A-B)-|C|(\partial(\frac{A-B}{P_{WMQMR}})/\partial\theta)\right]^2}{(A+B)[(A-B)^2+4|C|^2]}.\end{aligned} \tag{27}$$

Eq.(26) has three parts. The first and the second part of it are the calculation result of the first part of Eq.(25). The third part is the second part of Eq.(25) which we will discuss in the following. Although here we are discussing the QFI of θ, not ϕ, we can still apply Eq.(11) and Eq.(13) in the **Methods** of the main text to calculate the eigenvalues and eigenvectors of ρ^{out} in Eq.(22) according to the WMQMR. So substituting the needed parameters of Eq.(24) into Eq.(11) and Eq.(13) in the **Methods**, we can get $\lambda_{1,2}=[(A+B)\pm\sqrt{(A+B)^2-4(A\times B-C\times D)}\,]/(2P_{WMQMR})$, $|\varphi_1\rangle=\cos\eta|0\rangle^{\otimes N}+\exp(i\xi_1)\sin\eta|1\rangle^{\otimes N}$ and $|\varphi_2\rangle=-\sin\eta|0\rangle^{\otimes N}+\exp(i\xi_2)\cos\eta|1\rangle^{\otimes N}$ from them. And then substituting these eigenvalues and eigenvectors into the second part of Eq.(25) we can get: $2\frac{(\lambda_1-\lambda_2)^2}{\lambda_1+\lambda_2}\left|\langle\varphi_1|\frac{\partial}{\partial\theta}|\varphi_2\rangle\right|^2+2\frac{(\lambda_2-\lambda_1)^2}{\lambda_1+\lambda_2}\left|\langle\varphi_2|\frac{\partial}{\partial\theta}|\varphi_1\rangle\right|^2=4\frac{(\lambda_1-\lambda_2)^2}{\lambda_1+\lambda_2}\left|\frac{\partial\eta}{\partial\theta}\right|^2$. And then we use Eq.(15) of the **Methods** to calculate $\left|\frac{\partial\eta}{\partial\theta}\right|$. In the basis of above analysis, we get the third part of Eq.(26). Thus we get Eq.(26) and we know that i, j, l_m, A, B, C and P_{WMQMR} in it have been defined in Eq.(24).

In the main text, F_{DN} and Fid_{DN} can be gotten easily just by substituting p_{1h}=p_{rh}= 0 $(h=1,\ldots,N)$ into F_{WMQMR} and Fid_{WMQMR}. Like the derivation of them, if we substitute p_{1h}=p_{rh}= 0 $(h=1,\ldots,N)$ into F^{θ}_{WMQMR}, i.e., Eq.(26) and Eq.(27), we can get F^{θ}_{DN} of general version and simplified version separately.

Numeric calculations show that the average QFI of θ of WMQMR is always lower than that of DN for anyone (or both) of p_{1h} and p_{rh} is(are) not zero(s), and for any r_h, θ and qubit number N, $(h=1,\ldots,N)$. This is because the average QFI of

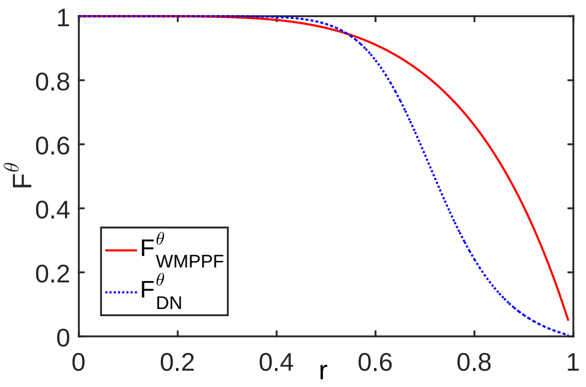

Figure 1. F^{θ} for WMPPF and DN with $N=10$, θ=$7\pi/8$. their upper limit is 1. It is clear that the former outperforms the latter when r is not small.

WMQMR is very sensitive to the probability, which can be reduced to lower than 1 if at least one of p_{1h} and p_{rh} is not zero. And this reduced probability will greatly decrease the average QFI. So we need not to discuss F^{θ}_{WMQMR}.

To DN case, calculations reveal that increasing qubit number N can delay the dissipation of the QFI of θ under the ADC. We draw Fig.(8) to display this conclusion in the main text.

Average QFI of weight factor for WMPPF. In this section we give the formula of average QFI of weight factor (i.e., amplitude parameter) θ for WMPPF, where the initial state is generalized GHZ state $|\Phi^{in}\rangle$ same as the former. This calculation is similar as the WMQMR case, and for simplicity we omit the derivation and only give the formula of the simplified version as previously stated:

$$
\begin{aligned}
F^{\theta}_{WMPPF} \overset{S}{=} & P_0\Big[\frac{(\frac{\partial\lambda_{01}}{\partial\theta})^2}{\lambda_{01}}+\frac{(\frac{\partial\lambda_{02}}{\partial\theta})^2}{\lambda_{02}}\Big]+P_0^2\sum_{k=1}^{N-1}C_N^k\frac{[(\frac{|\alpha|^2}{P_0})']^2}{|\alpha|^2}(1-p)^N s^{N-k}r^k+P_0^2\frac{4[(\frac{|C_0|}{P_0})'(A_0-B_0)-|C_0|(\frac{A_0-B_0}{P_0})']^2}{(A_0+B_0)[(A_0-B_0)^2+4|C_0|^2]} \\
& +P_N\Big[\frac{(\frac{\partial\lambda_{N1}}{\partial\theta})^2}{\lambda_{N1}}+\frac{(\frac{\partial\lambda_{N2}}{\partial\theta})^2}{\lambda_{N2}}\Big]+P_N^2\sum_{k=1}^{N-1}C_N^k\frac{[(\frac{|\beta|^2}{P_N})']^2}{|\beta|^2}(1-p)^N r^{N-k}s^k+P_N^2\frac{4[(\frac{|C_N|}{P_N})'(A_N-B_N)-|C_N|(\frac{A_N-B_N}{P_N})']^2}{(A_N+B_N)[(A_N-B_N)^2+4|C_N|^2]} \\
& +\sum_{k=1}^{N-1}C_N^kP_{l_k}\Big[\frac{(\frac{\partial\lambda_{l_k1}}{\partial\theta})^2}{\lambda_{l_k1}}+\frac{(\frac{\partial\lambda_{l_k2}}{\partial\theta})^2}{\lambda_{l_k2}}\Big]+\sum_{k=1}^{N-1}C_N^kP_{l_k}^2\frac{[p^k(1-p)^{N-k}(\frac{|\alpha|^2}{P_{l_k}})'r^{N-k}+(1-p)^kp^{N-k}(\frac{|\beta|^2}{P_{l_k}})'r^k]^2}{p^k(1-p)^{N-k}|\alpha|^2r^{N-k}+(1-p)^kp^{N-k}|\beta|^2r^k} \qquad (28) \\
& +\sum_{k=1}^{N-1}C_N^{N-k}P_{l_k}^2\Big[\frac{p^k(1-p)^{N-k}[(\frac{|\alpha|^2}{P_{l_k}})']^2\sum_{m=1}^{N-k-1}C_{N-k}^m s^{N-k-m}r^m}{|\alpha|^2}\Big]+\sum_{k=1}^{N-1}C_N^kP_{l_k}^2\Big[\frac{(1-p)^kp^{N-k}[(\frac{|\beta|^2}{P_{l_k}})']^2\sum_{m=1}^{k-1}C_k^m r^{k-m}s^m}{|\beta|^2}\Big] \\
& +\sum_{k=1}^{N-1}C_N^kP_{l_k}^2\frac{4[(\frac{|C_{l_k}|}{P_{l_k}})'(A_{l_k}-B_{l_k})-|C_{l_k}|(\frac{A_{l_k}-B_{l_k}}{P_{l_k}})']^2}{(A_{l_k}+B_{l_k})^3}.
\end{aligned}
$$

Here the $\lambda_{f1,2}=[(A_f+B_f)\pm\sqrt{(A_f+B_f)^2-4(A_f\times B_f-C_f\times D_f)}\,]/(2P_f)$ $(f=0,N,l_k)$. And A_f, B_f, C_f, D_f and P_f $(f=0,N,l_k)$ are corresponding to the ones given in the first section of this text. And $(\ )'$ indicates taking the derivative of the content inside the $(\ )$ with respect to θ.

Many numeric calculations infer that F^{θ}_{WMPPF} can not protect the average QFI of θ for the state of $\theta\leq\pi/2$, including GHZ state ($\theta=\pi/2$, $\phi_0=0$), and can only protect that for generalized GHZ state under some very special conditions, i.e., $\theta>\pi/2$ (especially, it is close to π) and r is not small. We draw Fig.1 to show this character, and compare F^{θ} of WMPPF with that of DN for $N=10$, θ=$7\pi/8$. From it we can see that their upper limit is 1. It is clear that the former outperforms the latter when r is not small.
